# A Blind Circadian Clock in Cavefish Reveals that Opsins Mediate Peripheral Clock Photoreception

**DOI:** 10.1371/journal.pbio.1001142

**Published:** 2011-09-06

**Authors:** Nicola Cavallari, Elena Frigato, Daniela Vallone, Nadine Fröhlich, Jose Fernando Lopez-Olmeda, Augusto Foà, Roberto Berti, Francisco Javier Sánchez-Vázquez, Cristiano Bertolucci, Nicholas S. Foulkes

**Affiliations:** 1 Department of Biology and Evolution, University of Ferrara, Ferrara, Italy; 2 Institute of Toxicology and Genetics, Karlsruhe Institute of Technology, Eggenstein, Germany; 3 Department of Physiology, Faculty of Biology, University of Murcia, Murcia, Spain; 4 Department of Evolutionary Biology “Leo Pardi,” University of Firenze, Firenze, Italy; University of Geneva, Switzerland

## Abstract

The circadian clock is synchronized with the day-night cycle primarily by light. Fish represent fascinating models for deciphering the light input pathway to the vertebrate clock since fish cell clocks are regulated by direct light exposure. Here we have performed a comparative, functional analysis of the circadian clock involving the zebrafish that is normally exposed to the day-night cycle and a cavefish species that has evolved in perpetual darkness. Our results reveal that the cavefish retains a food-entrainable clock that oscillates with an infradian period. Importantly, however, this clock is not regulated by light. This comparative study pinpoints the two extra-retinal photoreceptors Melanopsin (Opn4m2) and TMT-opsin as essential upstream elements of the peripheral clock light input pathway.

## Abstract 2

Evolution during millions of years in perpetual darkness leads to mutations in non-visual opsin genes (Melanopsin and TMT opsin) and an aberrant, blind circadian clock in cavefish.

## Author Summary

The circadian clock is a physiological timing mechanism that allows organisms to anticipate and adapt to the day-night cycle. Since it ticks with a period that is not precisely 24 h, it is vital that it is reset on a daily basis by signals such as light to ensure that it remains synchronized with the day-night cycle. The molecular mechanisms whereby light regulates the clock remain incompletely understood. Here we have studied a cavefish that has evolved for millions of years in the perpetual darkness of subterranean caves in Somalia. Like many other cave animals, these fish display striking adaptations to their extreme environment, including complete eye degeneration. We show that despite evolving in a constant environment, this blind cavefish still retains a circadian clock. However, this clock ticks with an extremely long period (nearly 47 h), and importantly it does not respond to light. We reveal that eye loss does not account for this “blind” clock. Specifically, mutations of two widely expressed non-visual opsin photoreceptors (Melanopsin and TMT opsin) are responsible for the blind clock phenotype in the cavefish. Our work illustrates the great utility of cavefish for studying the evolution and regulation of the circadian clock.

## Introduction

The circadian clock is a highly conserved, physiological timing mechanism that allows organisms to anticipate and adapt to daily environmental changes and it is synchronized primarily by light. In mammals, intrinsically photosensitive retinal ganglion cells serve as the principal circadian photoreceptors [1]. In non-mammalian vertebrates, photoreceptors located outside of the retina (in the pineal complex and in the deep brain) have also been implicated in the regulation of the circadian timing system [2]. At the core of the vertebrate circadian clock is a transcription translation feedback loop mechanism composed of activator and repressor clock proteins [3]. Light-induced expression of certain clock genes represents a key step in the relay of lighting information to the core clock machinery [4],[5].

The zebrafish (*Danio rerio*) represents a fascinating model to study the mechanisms whereby light regulates the clock. The “peripheral” clocks in most zebrafish tissues and even cell lines are entrained by direct exposure to light [6]. However, fundamental questions concerning the identity of the widely expressed photoreceptor molecules and how they signal to peripheral clocks remain unanswered. To date, a set of widely expressed opsins, one cryptochrome homolog, and flavin-containing oxidases have all been implicated as candidate peripheral photoreceptors [7]–[9].

In certain extreme environments such as caves some fish species have remained completely isolated from the day-night cycle for millions of years [10]. They show convergent evolution, sharing a range of striking physical “troglomorphic” properties including notably degeneration of the eyes during early development. However, many aspects of cavefish biology still remain incompletely understood. Does evolution in constant darkness lead to loss of other aspects of photoreceptor function including regulation of peripheral circadian clocks by light? Furthermore, do these remarkable animals even retain normal circadian clocks?

In this report we explore the circadian clock and its regulation by light in *Phreatichthys andruzzii*, a Somalian cavefish that shows an extreme troglomorphic phenotype. We compare the circadian clock mechanism of this cavefish with that of the zebrafish with the goal of identifying key components of the light input pathway. We reveal that *P. andruzzii* possesses a clock that is entrained by periodic food availability, displays a long infradian period, and lacks temperature compensation. However, importantly, this cavefish clock is no longer entrainable by light. Strikingly, in the cavefish we encounter mutations in the candidate non-visual photoreceptors Melanopsin (Opn4m2) and TMT (teleost multiple tissue)-opsin and we provide direct evidence for a light-sensing function of these non-visual opsins in the regulation of vertebrate peripheral clocks.

## Results

### Exploring the Circadian Clock in the Cavefish *Phreatichthys andruzzii*

**Figure 1:**
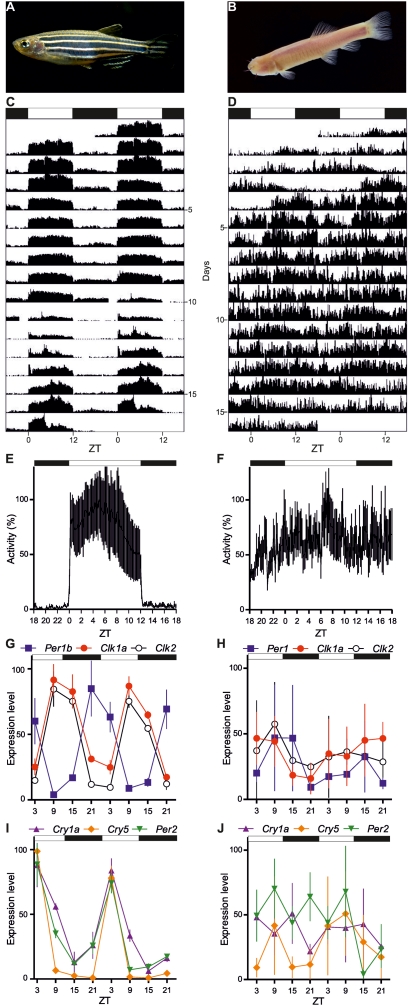
Lack of rhythmicity in *P. andruzzii* under light dark cycles. Adult zebrafish (A) and *P. andruzzii* (B). (C,D) Representative actograms of zebrafish (C) and cavefish (D) maintained under LD conditions (each cycle: 12 h light, 12 h dark) and fed randomly. Records are double plotted on a 48 h time scale to aid interpretation; the *y*-axis progresses in single days with each day being plotted twice (day 1 on the right side is repeated on day 2 on the left side). The activity was binned every 10 min, the height of each point representing the number of interruptions of the infrared light beam. (E,F) Mean waveforms of zebrafish (E) and cavefish (F) are represented. Each point in the mean waveform has been calculated as the mean ± SEM from 10 min binned data across all the experimental days shown (*n* = 16) on each actogram and for all experimental aquaria (*n* = 6 for zebrafish and *n* = 6 for cavefish). For χ^2^ periodogram analysis (confidence level, 95%), see Figure S1. (G–J) Quantitative RT-PCR analysis of endogenous clock gene expression for 2 consecutive days in adult zebrafish and *P. andruzzii* fins (G,I and H,J, respectively; *n* = 6 per time point) exposed to 12∶12 LD cycles. For all panels, each point represents the mean ± SEM. In *P. andruzzii*, neither clock-regulated (*Per1* (blue), *Clk1a* (red), and *Clk2* (black) (H)), nor light-regulated genes (*Cry1a* (purple), *Cry5* (orange), and *Per2* (green) (J)), show significant cycling (*p*>0.1) compared with high amplitude rhythms for the same genes observed in the zebrafish (*p*<0.0001) (panels G and I). White and black bars represent light and dark periods, respectively. On the *y*-axes are plotted relative expression levels, while on the *x*-axes time is expressed as zeitgeber time (ZT, where ZT0 represents lights on).

We chose to study a species of cavefish expressing an extreme “troglomorphic” phenotype, *P. andruzzii* (Figure 1B). This Somalian cavefish evolved from surface dwelling ancestors, isolated in a totally dark environment beneath the desert at a nearly constant temperature for 1.4–2.6 million years [10], approximately 1 million years longer than the well-studied cavefish *Astyanax mexicanus* [11]. *P. andruzzii* shows total eye degeneration, no scales, and complete depigmentation [12].

**Figure 2:**
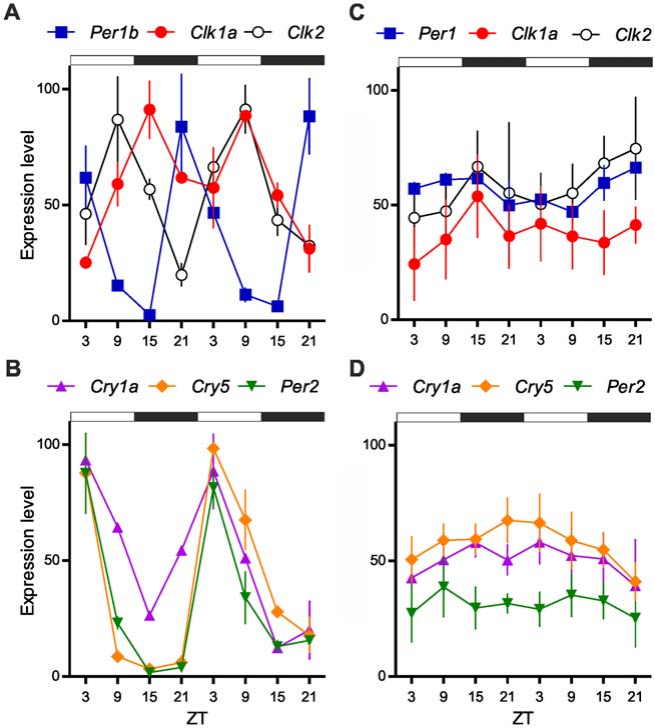
Lack of rhythmic clock gene expression in *P. andruzzii* cell lines exposed to LD cycles. Quantitative RT-PCR analysis of clock and light-regulated clock gene expression in zebrafish (A,B) and cavefish (CF; C,D) cell lines. The presentation, color code for the plotted data, and the experimental conditions are identical to Figure 1G–J. Each point represents the mean ± SEM.

As a first step we wished to explore the circadian clock and its regulation by light in this cavefish species. We initially measured locomotor activity in cavefish exposed to a 12∶12 light-dark (LD) cycle (Figures 1D,F and S1B). We documented striking arrhythmic locomotor activity that contrasts with the clear rhythmic, diurnal pattern observed for zebrafish under the same conditions (Figures 1A,C,E and S1A). In order to explore at the molecular level this lack of behavioural rhythmicity in cavefish, we next subcloned a set of *P. andruzzii* clock gene homologs with the aim of examining their expression pattern under LD cycles (Table S1). These genes were selected since their zebrafish counterparts are either clock- or light-regulated. Sequence analysis confirmed a close similarity between zebrafish and *P. andruzzii* genes (Table S2 and Figure S2), consistent with both species belonging to the Cyprinidae family. We measured the expression of a subset of clock-regulated (*Clk1a*, *Clk2*, *Per1*, *Per1b*) and light-regulated genes (*Per2*, *Cry1a*, and *Cry5*) [4],[5],[8],[13],[14] *in vivo* in adult tissues and in whole larvae from both species (Figure 1G–J and Figure S3). In the zebrafish, in agreement with previous reports, exposure to a LD cycle results in robust rhythmic expression of these genes (Figure 1G,I and Figure S3A,B,E) [6],[14]. Remarkably, arrhythmic gene expression was encountered in cavefish tissues and larvae (Figure 1H,J and Figure S3C,D,F,G), even during the first day of life when eye rudiments still persist [12]. Importantly, these clock gene expression patterns are consistent with the behavioural activity profiles observed under LD cycles for both species. Circadian clocks are encountered in most vertebrate cell types and even in cell cultures *in vitro*. Furthermore, the persistence of circadian clocks *in vitro* has enabled many more detailed mechanistic studies. Therefore, we established cell lines from adult cavefish caudal fins (CF cells) to test whether *P. andruzzii* cells *in vitro* also lack rhythmic gene expression under LD conditions. Again we observed arrhythmic clock gene expression in *P. andruzzii* cells (Figure 2C,D) that contrasts with the robust rhythms documented in zebrafish cells (Figure 2A,B) [6],[15],[16]. Thus, we failed to detect rhythmic clock gene expression *in vivo* or *in vitro* in *P. andruzzii* under LD cycles. So our data reveal that either *P. andruzzii* lacks the circadian clock itself or it has a clock lacking a functional light input pathway.

**Figure 3:**
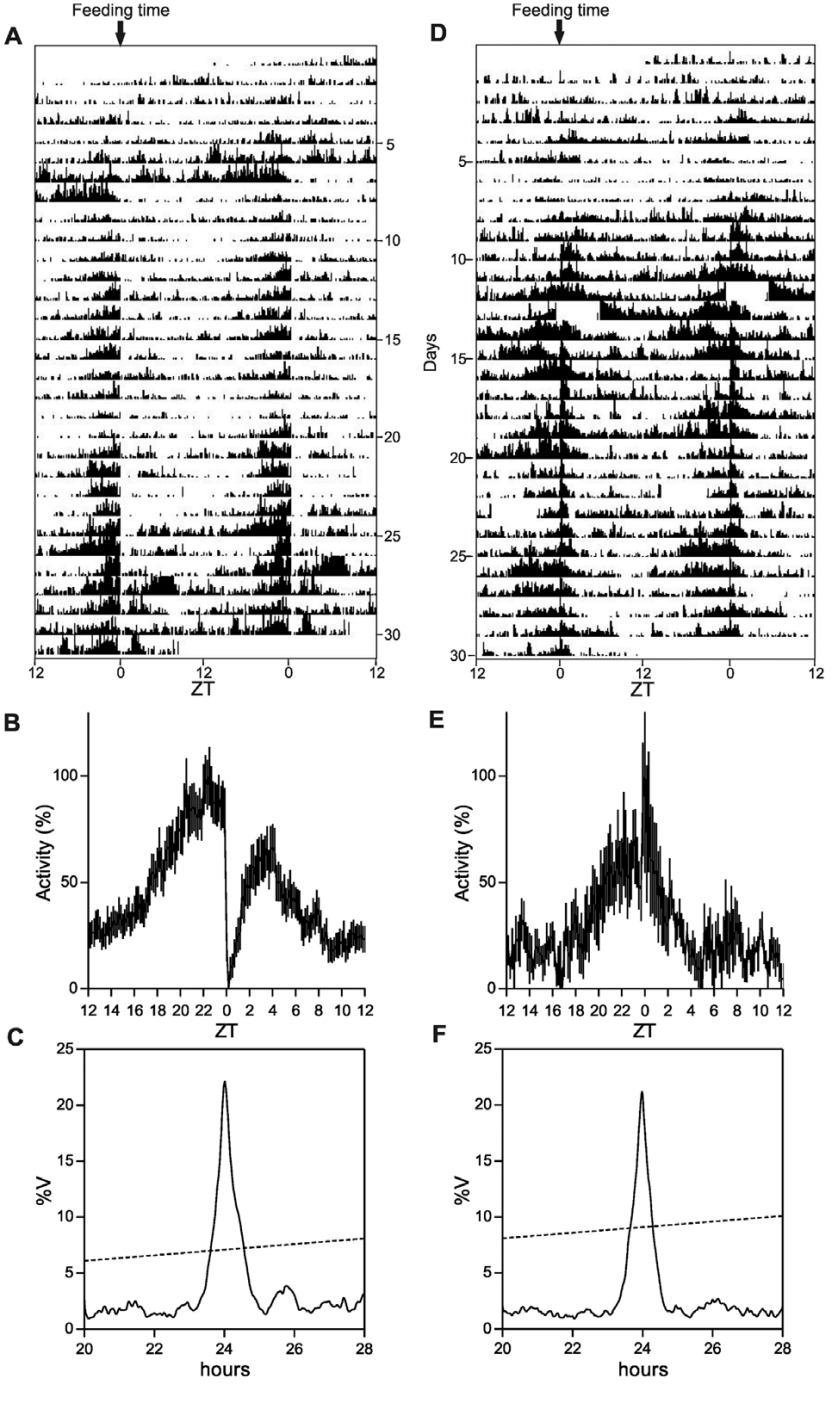
Behavioral entrainment by periodic food availability. Representative actograms of zebrafish (A) and cavefish (D) maintained under constant darkness and fed once a day at a fixed time (ZT = 0). Feeding time is indicated by the black arrow at the top of each actogram (for more details on actogram representation, see legend; Figure 1). (B,E) Mean waveforms of zebrafish (B) and cavefish (E) are represented. Each point in the mean waveform has been calculated as the mean ± SEM from 10 min binned data across all the experimental days (*n* = 30) shown on each actogram and all experimental aquaria (*n* = 5 for zebrafish and *n* = 3 for cavefish). (C,F) χ^2^ periodogram analysis (confidence level, 95%) for zebrafish (C) and cavefish (F) actograms. The periodogram indicates the percentage of variance (%V) of the rhythm explained by each analyzed period within a range of 20–28 h. The sloped dotted lines represent the threshold of significance, set at *p* = 0.05. Periodogram analysis showed the presence of behavioral activity rhythms synchronized to the 24 h feeding cycles.

**Figure 4:**
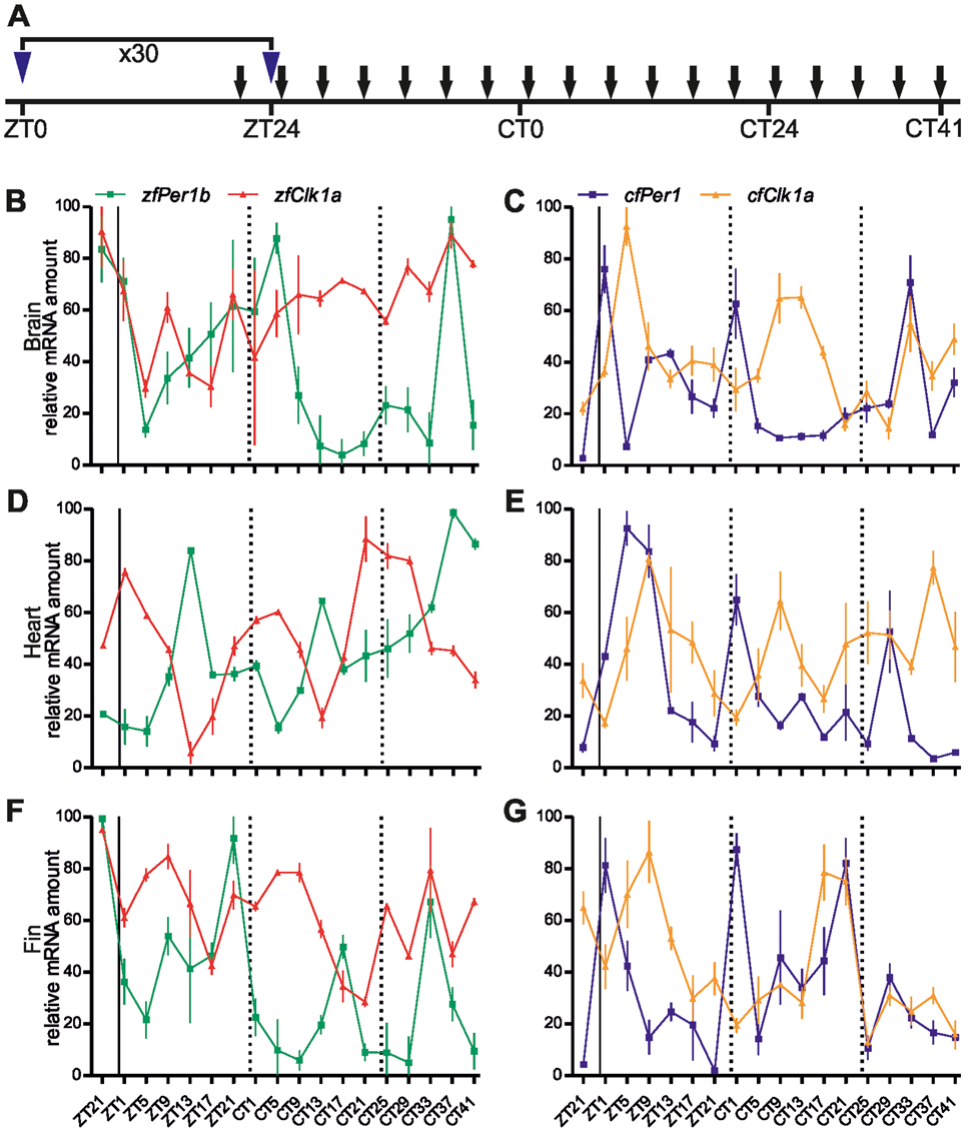
A non-photic zeitgeber entrains the cavefish clock. (A) Adult zebrafish and cavefish were fed once per day (at ZT0) for 30 d (dark blue arrowheads). Fish were then starved and after 24 h, brains, hearts, and fins were harvested each 4 h (*n* = 4) during the last day of food entrainment and the first 2 d of fasting (black arrowheads). (B–G) Real time PCR analysis of rhythmic endogenous *Clk1a* (red, orange) and *Per1* (green, dark blue) expression in the brain (B,C), heart (D,E), and fin (F,G) of zebrafish (B, D, and F) and cavefish (C, E, and G). Time is expressed as ZT or Circadian Time (CT) during starvation. In each panel, a solid, vertical line (at ZT0) indicates the last feeding time. Subsequently during starvation, the two vertical dotted lines (at CT0 and CT24) denote when the feeding would normally have occurred according to the previous regular feeding regime. Each point represents the mean ± SEM. In all cavefish tissues tested (C,E,G) robust circadian rhythms of *Clk1a* and *Per1* expression were observed (*p*<0.01). Rhythmic clock gene expression was also observed in zebrafish heart (D) and fin (F) (*p*<0.01). However, in the zebrafish brain (B) only *Per1b* (green trace) was rhythmically expressed (*p*<0.01). See also Figure S4.

To distinguish between these two hypotheses, we first assessed whether the cavefish circadian clock could be entrained by an alternative environmental time signal (*zeitgeber*), periodic food availability. Adult cavefish and zebrafish were fed once at the same time each day for one month under constant dark conditions, and during this period, locomotor activity was measured (Figure 3). For both species we observed food anticipatory activity (FAA, [17]), a characteristic increase in locomotor activity encountered a few hours prior to mealtime (Figure 3A,B,D,E) and a strong entrainment of rhythmic locomotor activity (Figure 3C,F). FAA is indicative of regulation by a food entrainable oscillator (FEO, [18]); thus we tested circadian clock gene expression in various tissues of both species during the final day of food entrainment and then two days of fasting under constant conditions (Figure 4A). Consistent with previous results [19], rhythmic clock gene expression (*Clk1a* and *Per1b*) was observed in zebrafish brain, heart, fin, and liver (Figures 4B,D,F and S4A), with the only exception of *Clk1a* in the zebrafish brain (Figure 4B, red trace). However, in all cavefish tissues including the brain, robust circadian rhythms of both *Clk1a* and *Per1* expression were observed (Figures 4C,E,G and S4B). In both species, differences in the phase and amplitude of rhythmic expression for each gene were observed between different tissues. Importantly, these results point to *P. andruzzii* having a functional clock that is entrainable by feeding but not by LD cycles. This contrasts with the situation in zebrafish where both light- and food-entrainable oscillators are present.

**Figure 5:**
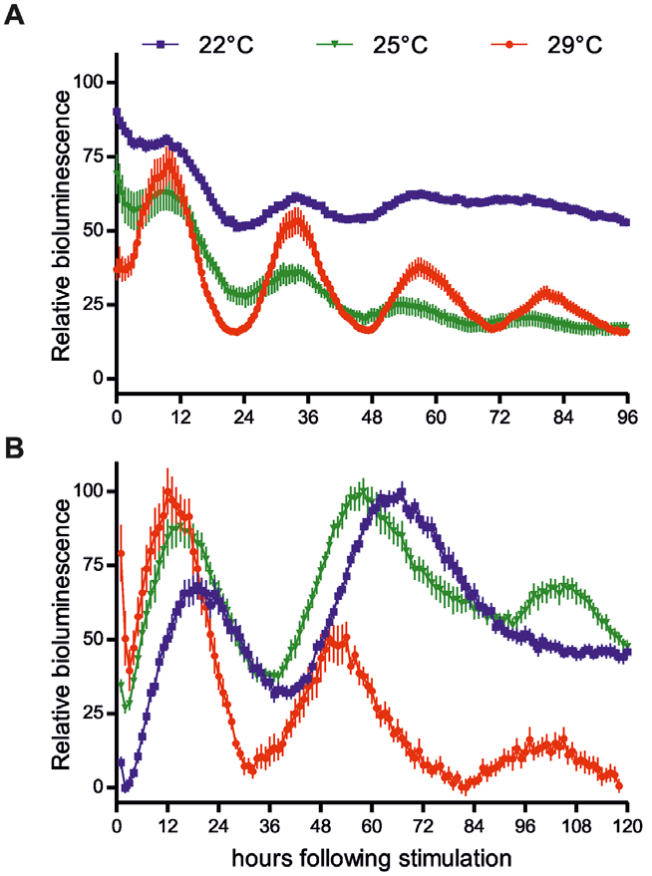
Reduced temperature compensation of the *P. andruzzii* clock. Following transfection with *zfper1b-Luc*, zebrafish (A) and CF (B) cells were transiently treated for 30 min with dexamethasone and then incubated either at 22°C (blue), 25°C (green), or 29°C (red). Dexamethasone induces rhythmic clock gene expression in cells at each of the three temperatures (*p*<0.0001). While in zebrafish cells (A) the period remains relatively constant with changes in temperature (Q_10_≈1), CF cells (B) showed a marked shortening of period length with increasing temperature indicating a poorly temperature compensated clock (Q_10_≈1.35) (for calculations, see Materials and Methods). Each point represents the mean ± SEM.

We also tested the effect of alternative zeitgebers on clock gene expression in cavefish CF cells. Transient treatments with glucocorticoids are widely used to induce rhythmic gene expression in cultured cells [20]. CF cells transfected with a clock-regulated zebrafish reporter construct (*zfPer1b-Luc*) [13] were treated transiently with 100 nM dexamethasone, an agonist of the glucocorticoid receptor (Figure S5; Figure 5B, green trace) [20]. Surprisingly, this induced a bioluminescence rhythm in cavefish cells that persisted for almost three cycles with an extremely long period (τ = 43 h at 25°C). This contrasts with the circadian bioluminescence rhythm observed upon dexamethasone treatment of zebrafish cells (τ = 24.2 h at 25°C) (Figure 5A, green trace). These results reveal the existence of an abnormal circadian clock in *P. andruzzii* that displays an infradian period under constant conditions. We next wished to determine whether other circadian clock properties are abnormal in the cavefish. One highly conserved feature of the circadian clock is that its period remains relatively constant over a physiological range of temperatures (so-called temperature compensation) [21]. Thus, we measured the period of rhythms induced by dexamethasone pulses in CF and zebrafish cells held at two additional constant temperatures 22°C and 29°C (Figure 5). With an increase in temperature the period length of the cavefish clock decreased significantly (τ = 47 (22°C), 43 (25°C), and 38 h (29°C)) revealing reduced temperature compensation with Q_10_≈1.35, while in zebrafish cells, as expected, a relatively constant period was observed (τ = 23.6 (22°C), 24.2 (25°C), and 24.6 (29°C)) h, respectively (Q_10_≈1 [22]). Thus, together our results indicate that *P. andruzzii* possesses a circadian timing system with an aberrant core clock mechanism.

Could the discrepancy between the striking infradian period of the *P. andruzzii* clock (τ>30 h) and the period of the LD cycle (T = 24 h) have been the origin of the observed arrhythmicity under LD cycles? In such a scenario, the cavefish clock might still be entrainable by light. In zebrafish cell lines, exposure to brief light pulses results in advances or delays in pre-existing clock gene expression rhythms depending on the precise time when the cells are illuminated [13]. Thus, we wished to test whether light is able to regulate the cavefish clock in a similar manner. We synchronized rhythmic clock gene expression in CF cells by dexamethasone treatment and then exposed the cells to a 15 min light pulse at five different time-points distributed throughout the 43 h cycle (Figure S6) [13],[23]. None of the light pulses changed either the phase or levels of rhythmic clock gene expression. Hence, we conclude that *P. andruzzii* indeed possesses a truly blind clock. Thus, this cavefish represents a powerful complementary model for exploring the function of the light input pathway in vertebrates.

### Defining Molecular Defects in the Light Input Pathway of *P. andruzzii*

**Figure 6:**
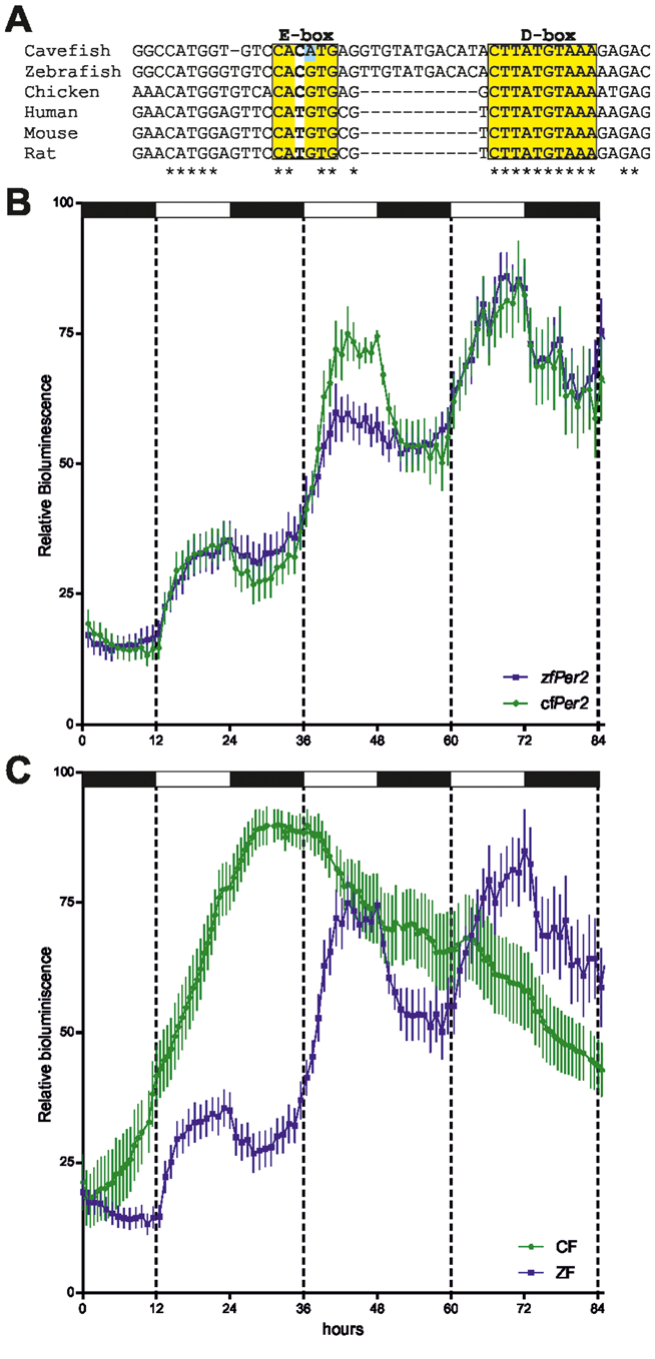
The defect in the cavefish light input pathway lies upstream of the *period2* gene promoter. (A) Alignment of the cavefish *period2* promoter LRM with the corresponding sequence from the zebrafish and other vertebrates [5]. Boxing highlights the highly conserved E- and D-box enhancers. Within each enhancer, yellow highlighted sequences are conserved in all species. Blue highlighting indicates the mutation in the cavefish E-box. Below, asterisks (*) indicate perfectly conserved sequences. (B) Zebrafish cells transfected with *cfPer2-Luc* (green trace) and *zfPer2-Luc* (blue trace) and exposed to LD cycles. Both constructs were rhythmically expressed (*p*<0.0001). The strongest induction coincided with the onset of the light period. (C) In vivo luciferase assays of cavefish (CF, green trace) and zebrafish cells (ZF, blue trace) transfected with *zfPer2-Luc*. The absence of rhythmic reporter expression in cavefish cells (*p*>0.1) contrasts with high amplitude rhythms observed in zebrafish cells (*p*<0.00001). The plotted values were calculated by subtracting the basal expression levels of the luciferase reporter. For each time point, mean ± SEM is plotted. White and black bars represent the light and dark periods, respectively.

Our systematic cloning and sequencing strategy failed to detect any mutations significantly affecting clock gene coding sequences (unpublished data). Light-induced transcription of clock genes represents a key step in photic entrainment of the zebrafish clock [4],[5]. Therefore, we speculated that mutations in promoter sequences of light-inducible clock genes could account for the cavefish blind clock phenotype. We have previously defined an essential light responsive module (LRM) containing E- and D-box enhancer elements in the zebrafish *per2* promoter [5]. In the LRM promoter region from the cavefish *per2* gene, we identified several base substitutions compared with the zebrafish sequence, including one in the E-box element (Figure 6A). We wished to test whether the cavefish LRM was still able to direct light-induced gene expression. With this aim we transfected zebrafish cells with a cavefish *per2* promoter driving luciferase expression (*cfPer2-Luc*) (Figure 6B, green trace). These cells exhibited robust light-driven reporter expression comparable to that of the zebrafish *per2* promoter (*zfPer2-Luc*) (Figure 6B, blue trace). Conversely, zebrafish *zfPer2-Luc* transfected into cavefish cells failed to show light-induced expression (Figure 6C, green trace). Together, these results indicate that mutations disrupting the cavefish light input pathway should lie upstream of directly light-regulated clock gene promoters.

### TMT-Opsin and Melanopsin Are Essential Photoreceptors for Peripheral Clocks

**Figure 7:**
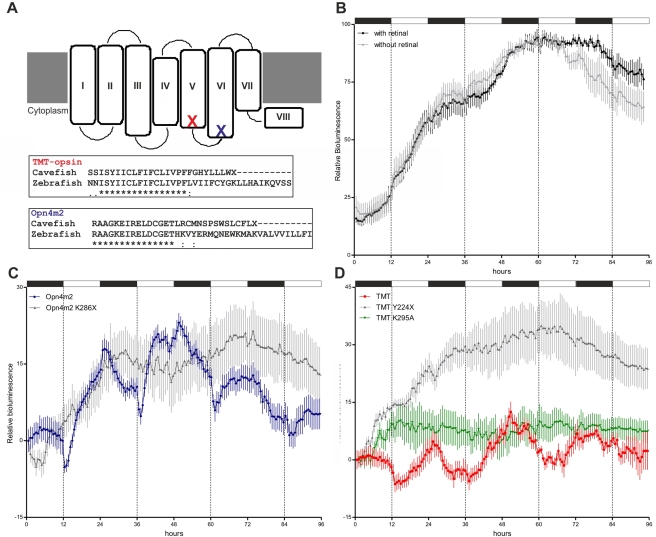
Rescue of light-induced gene expression in cavefish. (A) Schematic representation of the seven transmembrane domains of a generic opsin protein. Red and blue crosses denote the position of stop codons in cavefish TMT-opsin and Melanopsin (Opn4m2), respectively. Below, the C-termini of the truncated cavefish opsin proteins are aligned with the corresponding zebrafish sequences (asterisks (*) indicate identical aminoacids; periods (.), non-conservative aminoacids; and colons (:), conservative aminoacids. (B) Arrhythmic bioluminescence expression (*p*>0.5) of *zfper2-Luc* in CF cells under LD cycles in the presence (black) or absence (grey) of 100 nM 9-cis-/all-trans-retinal. (C–D) Rescued light-inducible expression in CF cells of *zfper2-Luc* co-transfected with zebrafish Opn4m2 (blue trace, C) and TMT-opsin (red trace, D) (*p*<0.0001). In contrast, expression of the zebrafish Opn4m2^K286X^ (C) and TMT^Y224X^ (D) carrying equivalent truncation mutations to the two cavefish opsins (grey traces) or TMT^K295A^ (with lysine 295 mutated to an alanine) (green trace) (D) failed to rescue rhythmic light induced *zfper2-Luc* expression (*p*>0.1). For each time point, mean ± SEM is plotted. White and black bars represent the light and dark periods, respectively.

Mutations affecting peripheral photoreceptors could also account for the blind cavefish clock. Although the identity of the teleost peripheral photoreceptor remains unclear, candidates include the opsins Melanopsin (Opn4m2) and TMT-opsin that are widely expressed in most tissues [9],[24]. Melanopsin was originally isolated from the photosensitive melanophores of *Xenopus* [25]. Subsequently, orthologs of Melanopsin were isolated from other non-mammalian vertebrates including zebrafish [26]. TMT-opsin was originally identified by virtue of its opsin sequence homology, and to date has only been isolated from teleosts [9]. We chose to clone and characterize these two opsins in the cavefish. We documented mRNA expression of both *Melanopsin* and *TMT-opsin* in various cavefish tissues including the CF cell line (Figure S7), consistent with previous results that revealed widespread expression for the zebrafish homologs [9],[24]. Furthermore, sequence alignment with other teleost homologs revealed strong conservation (unpublished data). Interestingly, however, premature stop-codons were encountered in the coding sequences of both TMT-opsin and Melanopsin (at the C-terminus of the 5^th^ transmembrane domain and the N-terminus of the 6^th^ transmembrane domain, respectively) (Figure 7A). These C-terminal truncations were the result of frame shift mutations in the two coding sequences (an insertion of one T in *TMT-opsin* at position +654 and deletion of one G in *Melanopsin* at position +842 relative to the ATG initiation codons of the zebrafish homolog sequences). Both opsin mutations would be predicted to eliminate binding of the essential chromophore, retinaldehyde, that normally occurs at the 7^th^ transmembrane domain. Therefore, we decided to test whether ectopic expression of the zebrafish homologs of these two opsins would rescue light-inducibility of a *zfPer2-Luc* reporter in cavefish cells. Simply supplementing the culture medium with retinaldehyde failed to induce rhythmic expression of *zfPer2-Luc* (Figure 7B). Strikingly, upon cotransfection with single opsin expression vectors, *zfPer2-Luc* was robustly induced during the light phase and subsequently decreased during the dark phase (Figure 7C, blue trace and 7D, red trace). In contrast, expression in cavefish cells of zebrafish Melanopsin and TMT-opsin carrying mutations introducing premature stop codons equivalent to the two cavefish opsins (zfOpn4m2^K286X^ and zfTMT^Y224X^) failed to rescue light inducible *zfper2-Luc* expression (Figure 7C and D, grey traces). These data are consistent with the predicted loss of the chromophore binding domains in the cavefish opsins. Furthermore, our results constitute strong evidence that Melanopsin and TMT-opsin indeed function as peripheral clock photoreceptors.

While Melanopsin represents a well-characterized photoreceptor [25],[26], until now TMT-opsin has been implicated as a photopigment based only upon sequence homology [9]. The cavefish represents a powerful model to confirm that TMT-opsin indeed functions as a photopigment. In all opsins a highly conserved lysine residue within the 7^th^ transmembrane domain provides a Schiff base linkage with the chromophore retinal that is critical for phototransduction [27]. In zebrafish TMT-opsin, a lysine residue at position 295 would be predicted to fulfil this role [9]. To test this prediction, we mutated lysine 295 to an alanine (TMT^K295A^). Expression of TMT^K295A^ failed to rescue light-induced *zfper2-Luc* expression in cavefish cells (Figure 7D, green trace), thus strongly indicating that TMT-opsin indeed functions as an opsin photopigment.

**Figure 8:**
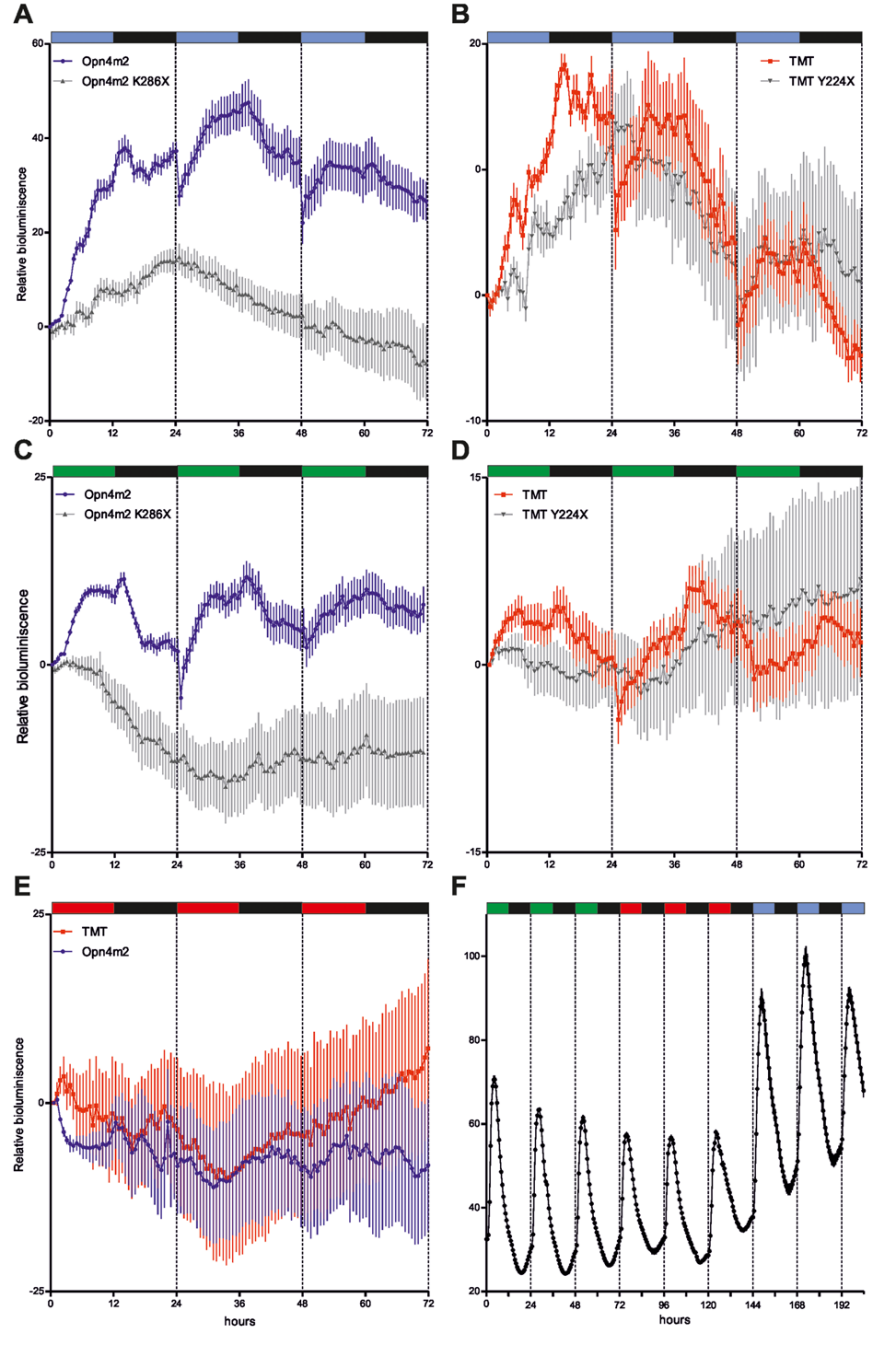
Rescue of light-induced gene expression in cavefish cells under different monochromatic light sources. Rescued rhythmic light-inducible expression of *zfper2-Luc* in CF cells transiently co-transfected with zebrafish Opn4m2 (blue trace, (A and C)) and TMT-opsin (red trace, (B and D)) exposed to blue (468 nm, A and B) or green (530 nm, C and D) light (*p*<0.0001). In contrast, no rescue was observed under red (657 nm) light (*p*>0.1) (E). The truncated zebrafish Opn4m2^K286X^ and TMT^Y224X^ (grey traces) failed to rescue rhythmic light-induced *zfper2-Luc* expression in all lighting conditions (*p*>0.2) (A–D). Interestingly, in zebrafish cells (PAC-2 −*1.7per2:Luc*) exposed to each of the same three monochromatic light sources, *zfper2-Luc* reporter expression is light-driven (*p*<0.0001) (black trace, (F)), with the strongest induction (+35% of peak values) observed under blue light. Above each panel, coloured and black bars indicate the types of light sources utilized and the duration of the light and dark periods. For each time point, mean ± SEM is plotted.

It is well established that the response of opsins to light is wavelength dependent. Melanopsin has been shown to respond preferentially to the blue region of the light spectrum [25], while the wavelength sensitivity of TMT-opsin is unknown. Are Melanopsin and TMT-opsin alone sufficient to account for the wavelength sensing properties of zebrafish peripheral clocks? As a first step towards addressing this key question, we repeated our cavefish *zfper2* expression rescue assay under three different monochromatic light sources (blue, green, and red, Figure S8). We then compared these results with the response of the *zfper2* promoter to the same light sources in zebrafish cells. Exposure of Melanopsin or TMT-opsin transfected cavefish cells to blue (468 nm) or green (530 nm) light is able to activate the *zfper2* promoter (Figure 8A–D). In contrast, no rescue was observed under red (657 nm) light (Figure 8E). However, exposure of zebrafish cells to these same monochromatic light sources revealed activation by all three light sources, with the strongest induction by blue (Figure 8F). These results confirm the wavelength sensitivity of Melanopsin [28] and provide the first evidence, to our knowledge, that TMT-opsin can respond to blue and green but not red wavelengths of light. The differences in the red light response of the *zfper2* promoter between the zebrafish cells and the rescued cavefish cell system point to the existence of additional peripheral photoreceptors. Importantly, comparable results were obtained in all our rescue experiments when we substituted *zfper2-Luc* for the minimal light-responsive promoter fragment of the zebrafish *per2* gene −*0.43per2:Luc* (unpublished data) [5]. This predicts that light-driven gene expression mediated by Melanopsin and TMT-opsin is dependent upon the LRM of the *per2* promoter.

## Discussion

In summary, during 1.4–2.6 million years of isolation from the day-night cycle, the evolution of the cavefish *P. andruzzii* has lead to an aberrant circadian clock. Contrary to the situation in most organisms, this clock is no longer entrained by light. Furthermore, upon exposure to alternative zeitgebers it cycles with a remarkably long infradian period and shows reduced temperature compensation. It is possible that this reflects progressive loss of a mechanism that provides no selective advantage for animals that live under constant darkness and temperature. In support of this hypothesis, one recent report has documented a severe attenuation of circadian clock rhythmicity in an arctic mammal naturally exposed to the extreme polar photic environment, the reindeer, *Rangifer tarandus* [29]. To further test this hypothesis, it will be fascinating to compare the circadian clock phenotype of *P. andruzzii* with that of other cavefish species representing the full range of troglomorphic phenotypes.

Our study reveals that a regular daily feeding time does entrain the cavefish clock. Interestingly, a comparison of the clock gene expression rhythms encountered in various tissues of the regularly fed cavefish reveals many differences in phase and amplitude between tissues. This situation is strongly reminiscent of the different patterns of cycling gene expression observed in individual light entrained peripheral clocks of zebrafish [30] and may reflect differences in the molecular regulatory networks associated with each tissue. Several reports point to the general importance of feeding entrainment for the circadian timing system in fish [17],[19]. It is tempting to speculate that food availability in the subterranean environment of this cavefish might indeed be periodic, and therefore a clock responding to and anticipating feeding time may confer a survival advantage. Interestingly, several lines of evidence point to the existence of a food-entrainable oscillator in vertebrates distinct from the light-entrainable oscillator, however the anatomical location and molecular mechanism remains unclear [18],[19],[31],[32]. In this regard, fish could emerge as powerful models for the investigation of food entrainment. A comparative study involving zebrafish that possess both light and food entrainable oscillators and *P. andruzzii* that retains only the food entrainable oscillator could provide important insight into the basis of the feeding entrainment mechanism in vertebrates.

By employing a comparative functional analysis involving zebrafish and *P. andruzzii*, we have been able to provide direct evidence that TMT-opsin and Melanopsin serve as peripheral tissue photoreceptors in teleosts. Furthermore, our results strongly indicate that the two opsins activate gene expression via the LRM promoter element that mediates light-driven *per2* expression [5]. Previously, the identity of the widely expressed photoreceptors that mediate the direct entrainment of peripheral clocks was unclear. In addition to opsins, other candidates include Cry4, a member of the cryptochrome family in zebrafish, and flavin-containing oxidases [7],[8]. It will now be important to re-evaluate the relative contribution of these other candidate photoreceptors to peripheral clock entrainment. Based on our study of the effects of different wavelengths of light on the induction of clock gene expression we predict that Melanopsin and TMT-opsin are not the only photoreceptors for peripheral clocks. This finding begs the question as to why several different photoreceptors might contribute to the photic entrainment properties of peripheral tissues. Changes in the photoperiod, intensity, as well as the spectral composition of sunlight represent reliable indicators of day-night as well as seasonal changes in the environment [33]. The presence of multiple photoreceptors, each one differentially extracting timing information from sunlight, could enable the circadian system to more reliably indicate the timing of dawn and dusk.

Finally from a broader perspective, in addition to displaying a unique and fascinating collection of adaptations to its extreme environment, we have demonstrated that *P. andruzzii* serves as a powerful complementary model to dissect the molecular pathways that respond to light.

## Materials and Methods

### Ethics Statement

The animal handling procedures and research protocols were approved by the University of Ferrara (Italy), University of Firenze (Italy), University of Murcia (Spain), and Karlsruhe Institute of Technology (Germany) Institutional Animal Care and Use Committees.

### Cavefish and Cavefish Cell Lines

*P. andruzzii* originally collected from Somalia (Figure S9) were maintained and bred at the University of Firenze, Italy. The cavefish were kept in darkness at a constant 27°C except during food administration and aquaria maintenance. Three times per week the fish were fed with frozen chironomid larvae. Fertilized eggs were collected every 30 min and aliquots of 10–20 eggs were transferred into 75 cm^2^ tissue culture flasks (BD GmbH). Flasks were sealed and submerged in a large volume, thermostatically controlled water bath (Tetra). From the third/fourth day after hatching, larvae were fed once a day.

Cavefish (CF) cell lines were derived from fin clips of adult fish and maintained using standard methods described elsewhere [34]. Cells were transiently transfected using FuGene HD reagent according to the manufacturer's recommendations (Roche) in the absence of serum. Rhythmic clock gene expression originally established by serum treatment during the seeding of the CF cells was resynchronized by a 30 min treatment with a range of dexamethasone (Sigma) concentrations from 50 nM to 1 µM (see Figure S5) [20]. Subsequently the dexamethasone-containing medium was replaced by fresh medium again lacking serum and containing luciferin.

### Lighting Conditions

To test for photic entrainment of rhythmic clock gene expression, cavefish and zebrafish adults and larvae as well as cell lines were maintained at 27°C under a 12∶12 LD cycle with a light intensity of 350 µW/cm^2^ (full-spectrum cool fluorescent tubes, Osram GmbH). For behavioural analysis, zebrafish and cavefish were maintained under full-spectrum cool fluorescent tube light sources with a light intensity of 20 µW/cm^2^. For monochromatic light sources, light-emitting diodes (LED, Kopa) sources were used (blue: λ_peak_ = 468 nm, green: λ_peak_ = 530 nm, red: λ_peak_ = 657 nm; white: 450 nm<λ<700 nm) (Figure S8). The light intensity of each LED light source was adjusted to ensure an equivalent number of photons were emitted from each source (1.42×10^18^±0.04×10^18^ photons/s/m^2^).

### Zebrafish and Zebrafish Cell Lines

All experiments using adult and larval zebrafish as well as the zebrafish cell lines AB9 [35], PAC-2, and a stable PAC-2 cell line expressing −*1.7per2:Luc* were performed using standard methods described elsewhere [5],[13]. Induction of rhythmic clock gene expression in zebrafish cells using transient dexamethasone treatment was performed as described for the cavefish CF cell line (see also Figure S5).

### Recording of Adult Locomotor Activity

Cavefish and zebrafish locomotor activity was registered continuously by means of an infrared photocell (E3S-AD62, Omron) placed at the aquarium wall, in the corner where food was provided. The photocell was placed 5 cm from the water surface and 20 cm from the bottom. The number of light-beam interruptions was counted and stored every 10 min by a computer connected to the photocell. The analysis of locomotor activity records, representation of actograms, and calculations of mean waveforms and χ^2^ periodograms (Sokolove-Bushell test) were performed using the chronobiology software *El Temps* (version 1.228).

### Cloning Cavefish cDNA Sequences

To obtain partial cDNA sequences, single-stranded cDNA was synthesized using SuperScript III Reverse Transcriptase (Invitrogen). Cavefish genes were amplified by PCR using Taq DNA Polymerase (Invitrogen) with primers designed by Primer3 software on the basis of sequence of the zebrafish homologs (Table S1). Bands of the predicted sizes were cloned into the pGEM-T Easy Vector (Promega). The cavefish gene cDNA fragments were sequenced (QIAGEN GmbH) and compared with the GenBank database by using the BLAST algorithm. Additional cDNA sequences were subsequently cloned using a 5′-3′SMART RACE cDNA amplification kit (BD Bioscience), and then coding sequences were deposited in GenBank (Table S2). By this approach, we cloned 13 clock genes (*Per1*, *Per2*, *Per3*, *Cry1a*, *Cry1b*, *Cry2a*, *Cry2b*, *Cry3*, *Cry4*, *Cry5 Clk1a*, *Clk1b*, *Clk2*) and 2 opsins (*Opn4m2*, *TMT-opsin*) from *P. andruzzii*.

### Phylogenetic Analysis

Sequences from the cavefish PER and CLK protein families were aligned with homologs from other teleost species (*Takifugu rubripes*, *Tetraodon nigroviridis*, *Danio rerio*, *Gasterosteus aculeatus*, and *Oryzias latipes*) [36],[37] using ClustalW. Alignments were manually verified and phylogenetic trees were generated using Neighbour-joining methods [38] with a complete deletion mode. Bootstrap tests were performed with 1,000 replications. Poisson correction distance was adopted and rates among sites were set as uniform. *Drosophila melanogaster* PER and CLK sequences were used as an out-group to root the trees.

### Gene Expression Analysis

Single-stranded cDNA was synthesized using SuperScript III Reverse Transcriptase (Invitrogen). Quantitative PCR was performed for *P. andruzzii* and zebrafish clock genes using the pairs of primers shown in Table S1. The StepOnePlus Real-Time PCR System (Applied Biosystems) was employed using SYBR-green-primer-master mix according to the manufacturer's recommendations with the following cycle conditions: 15 min at 95°C, then 40 cycles of 15 s at 95°C, and 30 s at 60°C. The relative levels for each RNA were calculated by the 2^−ΔΔCT^ method. Relative expression levels were normalized to *β-actin*. Each CT value is the mean of three biological replicates and each assay was performed a minimum of three times.

### Luciferase Reporter Constructs and Bioluminescence Assays

*zfPer1b-Luc* contains a promoter region extending 3.3 kb upstream of the 5′ end of the *Period1b* cDNA (equivalent to the *zfperiod4* promoter construct in [13]). The *zfPer2-Luc* reporter has been described previously as −*1.7per2:Luc* that contains a fragment of 1,571 bp upstream of the transcription start site and 129 bp of the 5′UTR of the zebrafish *Period2* gene [5]. In addition, the minimal light responsive *per2* promoter reporter construct −*0.43per2:Luc* was also tested in the cavefish cell rescue experiments. This construct contains 431 bp upstream of the transcription start site and 164 bp of the 5′UTR of the zebrafish *Period2* gene [5]. Both *zfPer2-Luc* reporters responded in an equivalent manner. *cfPer2-Luc* contains 876 bp upstream of the transcription start site and 112 bp of the 5′UTR of the cavefish *Period2* gene. All *in vivo* bioluminescence assays were performed as described previously [13],[34].

### Opsin Expression Vectors

The full-length zebrafish TMT-opsin (ENSDART00000081729) was amplified with the primers Fwd 5′-AATGGATTGCGGATTGGATCCATTGTGTCCAACTTG-3′ and Rev 5′-CTGCAGAATTCACTAGTGATTTCGCCTGTA-3′ resulting in the mutation of the ATG translation initiation codon sequence into TTC and the creation of BamHI and EcoRI restriction sites, respectively, for cloning into a modified pcDNA3.1(+) expression vector (Invitrogen) that incorporates an HA-Tag into the N-terminus of the expressed protein [39]. Similarly, the full length zebrafish *Opn4m2* (ENSDART00000018501) was amplified with the Fwd 5′-GCTCGGATCCGCCTTGAGCCATCACTCTTCA-3′ and Rev 5′-GCCCTCTAGACTCTTAGTTCCCTCCAAGCAA-3′ primers, thus mutating the ATG initiation codon into TTG as well as creating BamHI and XbaI sites, respectively, for cloning into the pcDNA3.1(+)HA-tag modified expression vector. PCR reactions were performed using the Perkin Elmer Gene Amp XL PCR kit according to the manufacturer's instructions. Construction of the expression vectors for zfTMT^K295A^ and the truncated forms zfOpn4m2^K286X^ and zfTMT^Y224X^ involved site-directed mutagenesis using the QuikChange MultiSite-Directed Mutagenesis Kit (Stratagene) according to the manufacturer's instructions. For the truncated forms, in the case of zfTMT-opsin the codon at position 224 (TAT, tyrosine) was mutated to a stop codon (TAA) while for Melanopsin (zfOpn4m2), the codon at position 286 (AAA, lysine) was mutated to a Stop codon (TAA). For the mutation of a key lysine in the 7^th^ transmembrane domain of zfTMT-opsin to an alanine residue, the codon sequence AAG was mutated to GCG. Following sequence analysis, the integrity and function of all opsin expression vectors was tested by transient transfection into a mammalian cell line and then Western blotting analysis using an anti-HA-tag specific antibody.

### Testing of Opsin Expression Constructs

Hepa1-6 cells (1×10^5^) (ATCC) were transfected with 2 µg of each HA-Tag opsin expression construct using the standard Promo-Fectin transfection protocol (PromoKine). Twenty-four hours after transfection, protein extracts were prepared according to a standard method [40], with some modifications. Cells were solubilized and incubated at 4°C in a mixture of equal volumes of TSA buffer (2 mM Tris-HCl, pH 8.0, 140 mM NaCl, 0.025% NaN_3_) and Lysis buffer (TSA buffer plus 2% Triton X-100, 5 mM iodoacetamide, 0.2 U/ml aprotinin, and 1 mM phenylmethylsulfonyl fluoride). After 1 h, 0.2 volumes of 5% sodium deoxycholate were added, and the mixture was incubated on ice for 10 min. The lysate was centrifuged at 2,800 g for 10 min at 4°C, and the supernatant was collected and stored at −80°C until use. Before electrophoresis in 10% or 15% SDS-polyacrylamide gels, the proteins were diluted in Laemmli buffer (final concentration 0.05 M Tris, pH 6.8, 2% SDS, 100 mM DTT, 10% glycerol, 0.05% bromophenol blue) without heating. Following transfer to nitrocellulose, the membranes were incubated with a high affinity anti-HA rat monoclonal antibody (clone 3F10, Roche) according to the manufacturer's instructions and the bound antibody was visualized using the ECL detection system (Amersham Biosciences) (Figure S10).

### Statistical Analysis

All the results were expressed as means ± SEM. Data were analyzed by one- or two-way analysis of variance (ANOVA) to determine significant differences using the software GraphPad Prism 4.0 (GraphPad Software Inc.). *p* values<0.05 were considered statistically significant. To evaluate the period length of gene expression, we measured the time span between two consecutive peaks. A trigonometric statistical model was applied to evaluate periodic phenomena. The single cosinor procedure [41] was used to define the main rhythmic parameters (circadian period and peak time).

Bioluminescence data were analyzed using Microsoft Excel or CHRONO software [13],[42]. Period estimates measured after 2 d in DD were made by linear regression following peak finder analysis with CHRONO. For Q_10_ temperature coefficient calculations, period length estimates for cells held at 22°C, 25°C, and 29°C were calculated as cycles per hour and then plotted against temperature. Linear regression analysis revealed a good fit to a straight line (cavefish R^2^ = 0.99; zebrafish R^2^ = 0.98). Mean period lengths at 22°C and 29°C were then substituted into the equation Q_10_ = (R_2_/R_1_)^10/(T2−T1)^, where R is rate and T is temperature.

### Genbank Accession Numbers

The sequences reported in this article are deposited in GenBank under accession numbers GQ404475–GQ404490 (see Table S2).

## Supporting Information

Figure S1 Periodogram analysis of behavioral activity in LD cycles. χ^2^ periodogram analysis (confidence level, 95%) for the zebrafish (A) and cavefish (B) actograms shown in Figure 1. The periodogram indicates the percentage of variance (%V) of the rhythm explained by each analyzed period within a range of 20–28 h. The sloped dotted lines represent the threshold of significance, set at *p* = 0.05. Periodogram analysis confirms that a behavioral activity rhythm is synchronized with the 24 h LD cycle in zebrafish but not in cavefish.
(TIF)

Figure S2 Phylogenetic analysis of cavefish clock genes. Comparison of PERIOD (top panel) and CLOCK (bottom panel) cavefish proteins with other published teleost homologs [36],[37] using phylogenetic tree analysis. This confirms the close similarity between zebrafish and *P. andruzzii* sequences.
(TIF)

Figure S3 Absence of rhythmic clock gene expression in cavefish brain and larvae. Quantitative RT-PCR analysis of endogenous clock gene expression under LD cycles in the whole brain of zebrafish (A,B) and cavefish (C,D) (*n* = 6 per time point) as well as in 5-d-old zebrafish larvae (E) and 1-d- or 4-wk-old cavefish larvae (F and G, respectively). Results are plotted as described in Figure 1. In the case of cavefish, no rhythmic expression was detected either in the brain (C,D; *p*>0.1), 1-d-old larvae which still retain eye rudiments (F, *p*>0.07), or even in larvae exposed for 4 wk to LD cycles (G, *p*>0.1).
(TIF)

Figure S4 Rhythmic clock gene expression in the liver following feeding entrainment. Real-time PCR analysis of rhythmic endogenous *Clk1a* (red trace) and *Per1b* (green trace) expression in the liver of zebrafish (A) and *Clk1a* (orange trace) and *Per1* (dark blue trace) expression in the cavefish liver (B). Time is expressed as ZT time or Circadian Time (CT) during starvation. In each panel, a solid, vertical line (at ZT0) indicates the last feeding time. Subsequently during starvation, the vertical dotted lines (at CT0 and CT24) denote when the feeding would normally have occurred according to the previous regular feeding regime. Each point represents the mean ± SEM. In both species robust circadian rhythms of clock gene expression were observed (*p*<0.01) (see also Figure 4).
(TIF)

Figure S5 Effects of different dexamethasone doses to synchronize rhythmic clock gene expression. Bioluminescence (cps) of cavefish cells transfected with the zebrafish reporter construct, *zfPer1b-Luc* and transiently treated with different dexamethasone concentrations (50 nM, 100 nM, 500 nM, and 1 µM). A control lacking dexamethasone (“Control,” black trace) was also included. All 4 dexamethasone treatments were able to advance by 5±0.5 h the phase of a pre-existing oscillation evident in the control cells. Serum treatment during the seeding of the cells is responsible for the original establishment of this oscillation (unpublished data). The vertical dotted and solid lines indicate the peaks of the control and dexamethasone pulsed cells, respectively.
(TIF)

Figure S6 Light pulses fail to shift the phase of a dexamethasone-entrained cavefish clock. (A) Five sets of CF cells were transiently transfected with the *zfPer1b-luc* reporter and then at 8 h intervals (at time points I–V) were transiently treated with 100 nM dexamethasone before being simultaneously exposed to a 15 min light pulse. (B–F) Resulting bioluminescence profiles of the five sets of cells, compared with non-light-pulsed controls. At each time point, the mean ± SEM is plotted. During the assay, cells were maintained at a constant temperature and in constant darkness. In each panel, colours of the bioluminescence traces match those in the experimental design (A). Pale coloured traces represent constant dark controls, while dark coloured traces represent the light pulsed sets of cells (except D, where the light pulsed trace is shown as yellow and the dark control trace is shown as olive). None of the light pulsed sets of cells show a significant difference in phase relative to their constant dark controls (*p*>0.4).
(TIF)

Figure S7 Tissue localization of *Melanopsin (Opn4m2)* and *TMT-opsin* in cavefish. RT-PCR results using brain, heart, muscle, fin, and CF cell RNA for the amplification of *Melanopsin*, *TMT-opsin*, and *β-actin* (as control). Equal volumes of the three PCR reactions performed for each tissue were subsequently mixed and then products were visualized by agarose gel electrophoresis. *Melanopsin* was expressed in all samples, whereas *TMT-opsin* was not found in the heart and the skeletal muscle (Sk. Muscle). A negative control lacking reverse transcriptase at the cDNA synthesis step was also included (RT (−)).
(TIF)

Figure S8 Irradiance curves for different monochromatic light sources. Light-emitting diode sources were used to produce white light (black trace) (450 nm<λ<700 nm) and monochromatic light in the blue (λ_peak_ = 468 nm), green (λ_peak_ = 530 nm), and red (λ_peak_ = 657 nm) region of the spectrum. The light intensity of each source was adjusted to ensure that a constant number of photons was emitted by each source (1.42×10^18^±0.04×10^18^ photons/s/m^2^).
(TIF)

Figure S9 Origin of *Phreatichthys andruzzii*. Adult cavefish were originally collected in the wild in the oasis of Bud-Bud (04°11′19″N–46°28′27″E) in the centre of the Somalian desert during several expeditions to Africa (1968–1982). Ancestors of *P. andruzzii* entered the large phreatic layers of the Somalian desert that developed in Eocene horizontal limestone formations at the end of the Pliocene (1.4–2.6 million years ago) and became isolated with the extinction of epigean sister species as the result of extreme climatic changes.
(TIF)

Figure S10 Expression of HA-tagged opsins in Hepa1-6 cells. Western blot analysis of cells transfected with HA-tagged expression vectors which encode (A) zfOpn4m2 (ca. 57 kDa), zfTMT-opsin (ca. 45 kDa), zfTMT^K295A^ (ca. 45 kDa), and (B) zfTMT-opsin^Y224X^ (ca. 27 kDa) and zfOpn4m2^K286X^ (ca. 34 kDa). The position of HA-immunoreactive opsin bands is indicated by arrowheads. The presence of doublet bands for some opsins may result from the denaturing conditions used to prepare the 7-transmembrane domain protein extracts for SDS PAGE analysis. Protein extracts prepared from cells transfected with the empty expression vector were loaded as negative controls.
(TIF)

Table S1 PCR primers. Summary of the sequences of forward (F) and reverse (R) PCR primers used to amplify the initial *P. andruzzii* cDNA fragments (based on evolutionary conserved *D. rerio* nucleotide sequences) as well as the cavefish and zebrafish sequence primers used for quantitative PCR analysis.
(DOC)

Table S2 *P. andruzzii* circadian clock cDNAs. Summary of the Genbank accession numbers for each of the *P. andruzzii* clock-related cDNAs cloned and sequenced. For each cDNA, the percentage of amino acid similarity with the zebrafish (*D. rerio*) homologs is also indicated.
(DOC)

## Abbreviations

CF: cavefish
Clk: Clock
Cry: Cryptochrome
CT: circadian time
DD: constant darkness
d-old: days old
LD: light/dark
LRM: light responsive module
per1b: period1b
per2: period2
TMT: teleost multiple tissue
UTR: untranslated region
wk-old: weeks old
zf: zebrafish
ZT: Zeitgeber time
