# Supplementary figures and images for: A Blind Circadian Clock in Cavefish Reveals that Opsins Mediate Peripheral Clock Photoreception
Source: PLoS Biol. 2011 Sep 6;9(9):e1001142. doi: 10.1371/journal.pbio.1001142 (PMC3167789; doi:10.1371/journal.pbio.1001142)

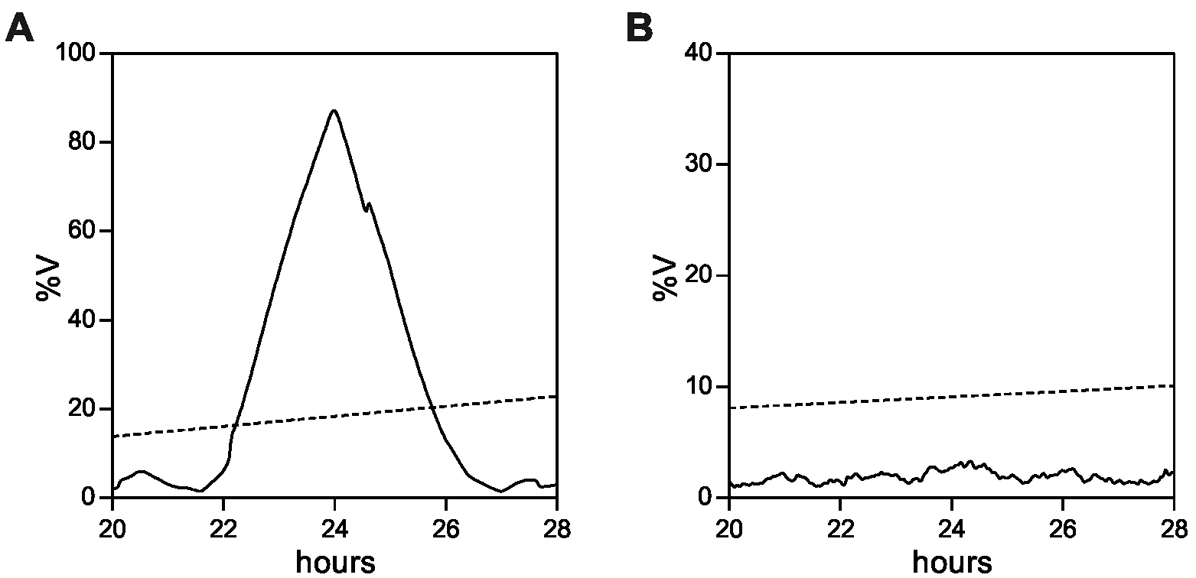

Supplement: Figure S1 — Periodogram analysis of behavioral activity in LD cycles. χ2 periodogram analysis (confidence level, 95%) for the zebrafish (A) and cavefish (B) actograms shown in Figure 1. The periodogram indicates the percentage of variance (%V) of the rhythm explained by each analyzed period within a range of 20–28 h. The sloped dotted lines represent the threshold of significance, set at p = 0.05. Periodogram analysis confirms that a behavioral activity rhythm is synchronized with the 24 h LD cycle in zebrafish but not in cavefish. (TIF) [file pbio.1001142.s001.tif]

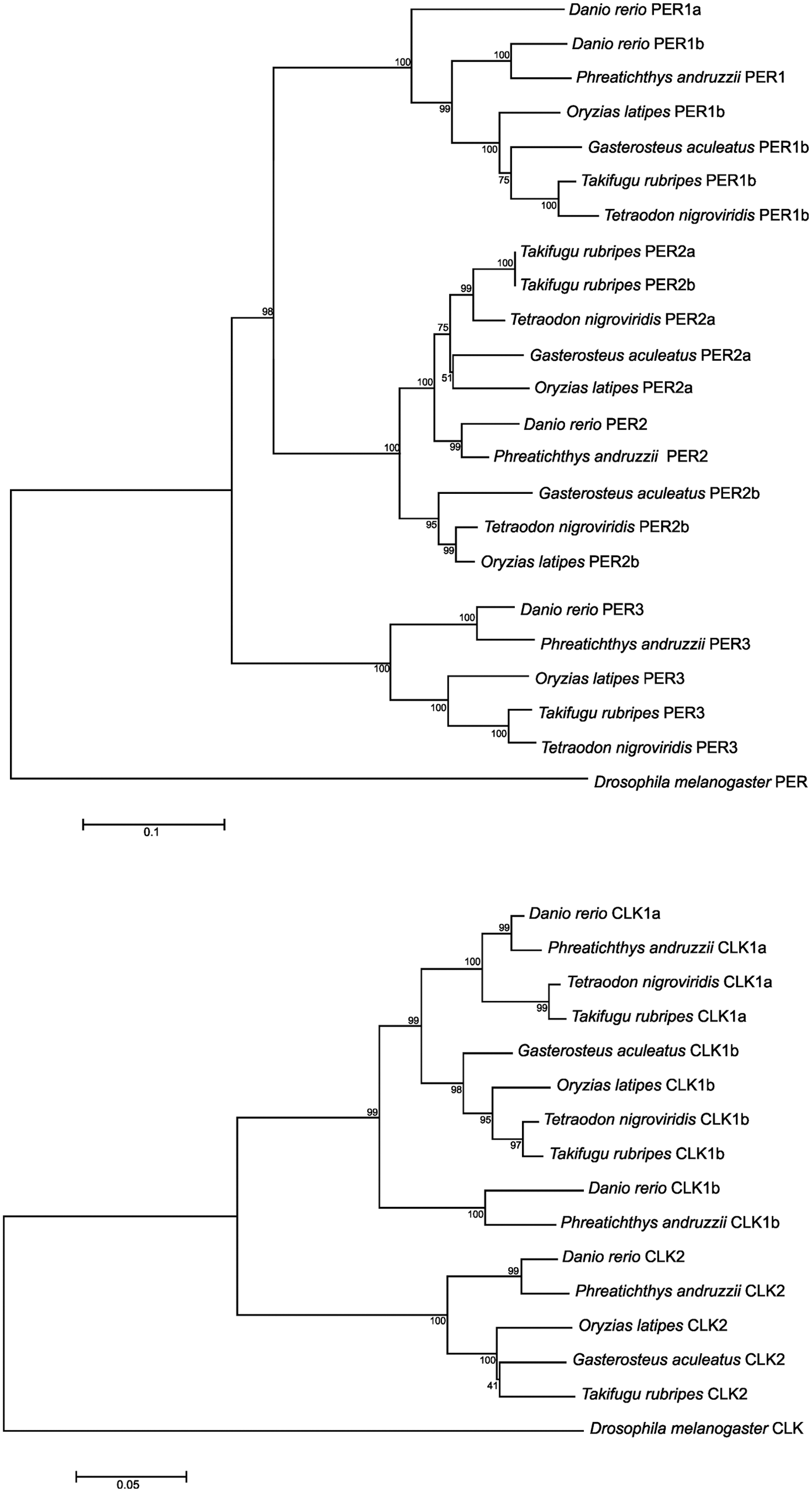

Supplement: Figure S2 — Phylogenetic analysis of cavefish clock genes. Comparison of PERIOD (top panel) and CLOCK (bottom panel) cavefish proteins with other published teleost homologs [36],[37] using phylogenetic tree analysis. This confirms the close similarity between zebrafish and P. andruzzii sequences. (TIF) [file pbio.1001142.s002.tif]

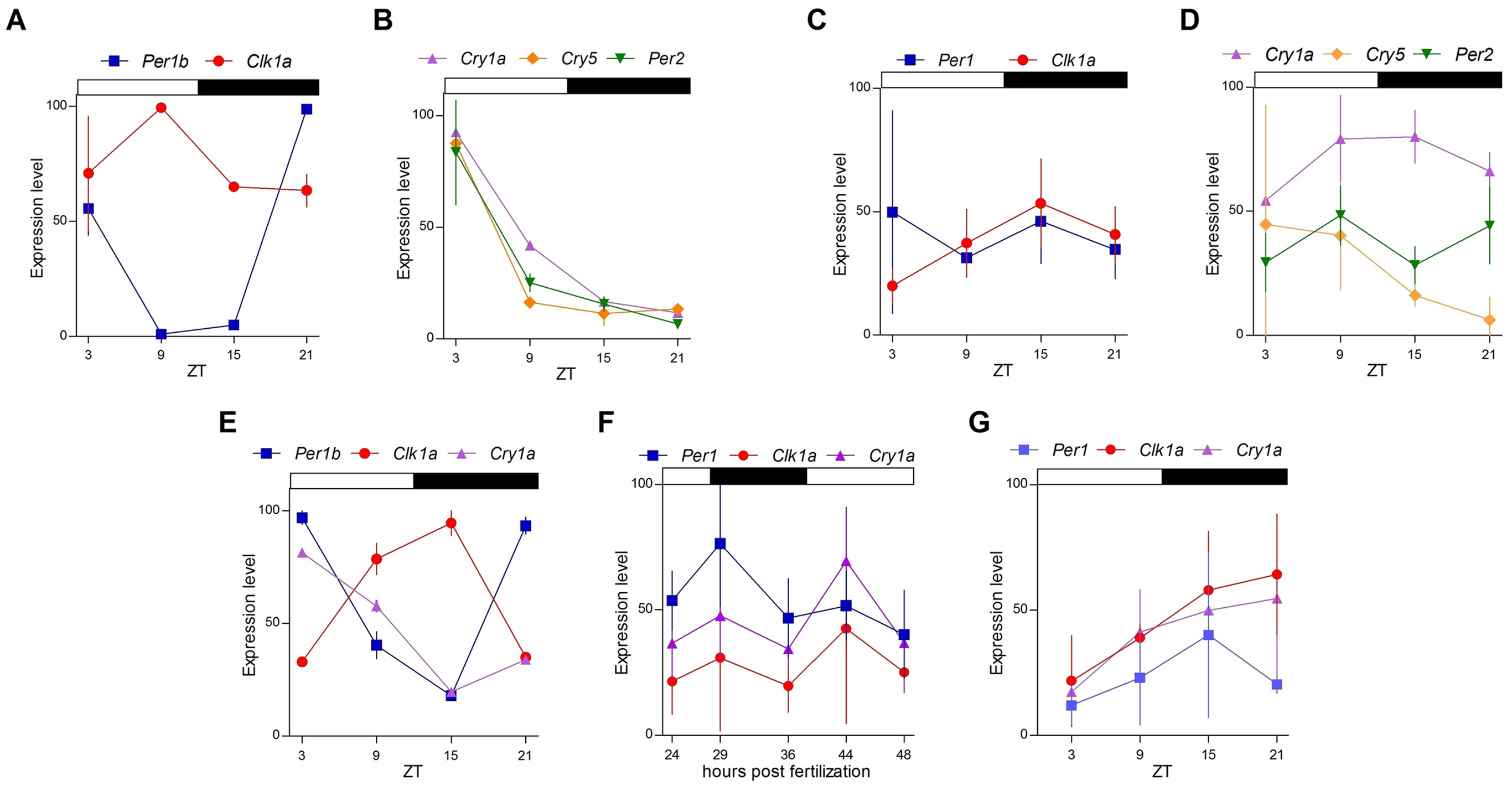

Supplement: Figure S3 — Absence of rhythmic clock gene expression in cavefish brain and larvae. Quantitative RT-PCR analysis of endogenous clock gene expression under LD cycles in the whole brain of zebrafish (A,B) and cavefish (C,D) (n = 6 per time point) as well as in 5-d-old zebrafish larvae (E) and 1-d- or 4-wk-old cavefish larvae (F and G, respectively). Results are plotted as described in Figure 1. In the case of cavefish, no rhythmic expression was detected either in the brain (C,D; p>0.1), 1-d-old larvae which still retain eye rudiments (F, p>0.07), or even in larvae exposed for 4 wk to LD cycles (G, p>0.1). (TIF) [file pbio.1001142.s003.tif]

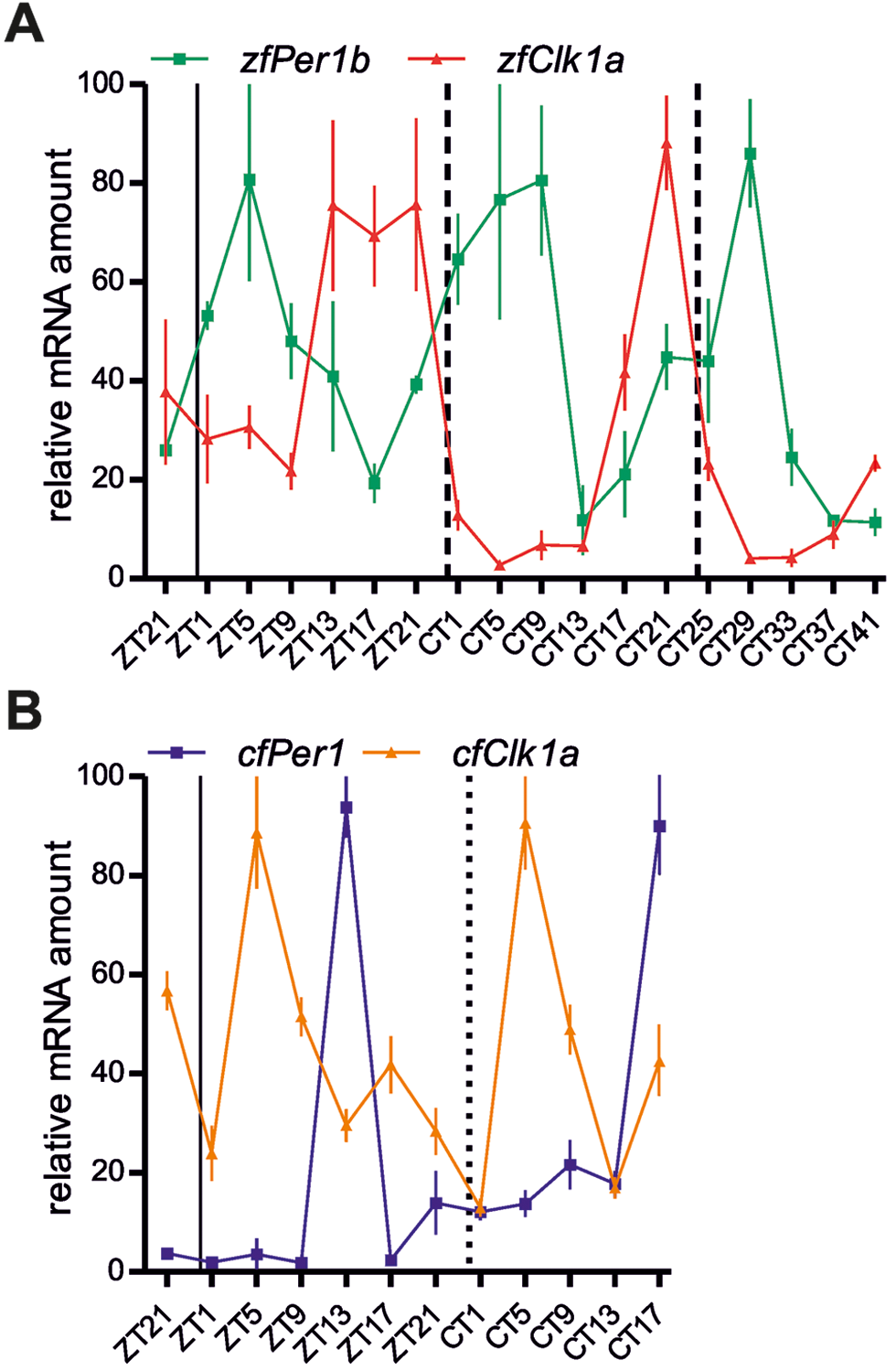

Supplement: Figure S4 — Rhythmic clock gene expression in the liver following feeding entrainment. Real-time PCR analysis of rhythmic endogenous Clk1a (red trace) and Per1b (green trace) expression in the liver of zebrafish (A) and Clk1a (orange trace) and Per1 (dark blue trace) expression in the cavefish liver (B). Time is expressed as ZT time or Circadian Time (CT) during starvation. In each panel, a solid, vertical line (at ZT0) indicates the last feeding time. Subsequently during starvation, the vertical dotted lines (at CT0 and CT24) denote when the feeding would normally have occurred according to the previous regular feeding regime. Each point represents the mean ± SEM. In both species robust circadian rhythms of clock gene expression were observed (p<0.01) (see also Figure 4). (TIF) [file pbio.1001142.s004.tif]

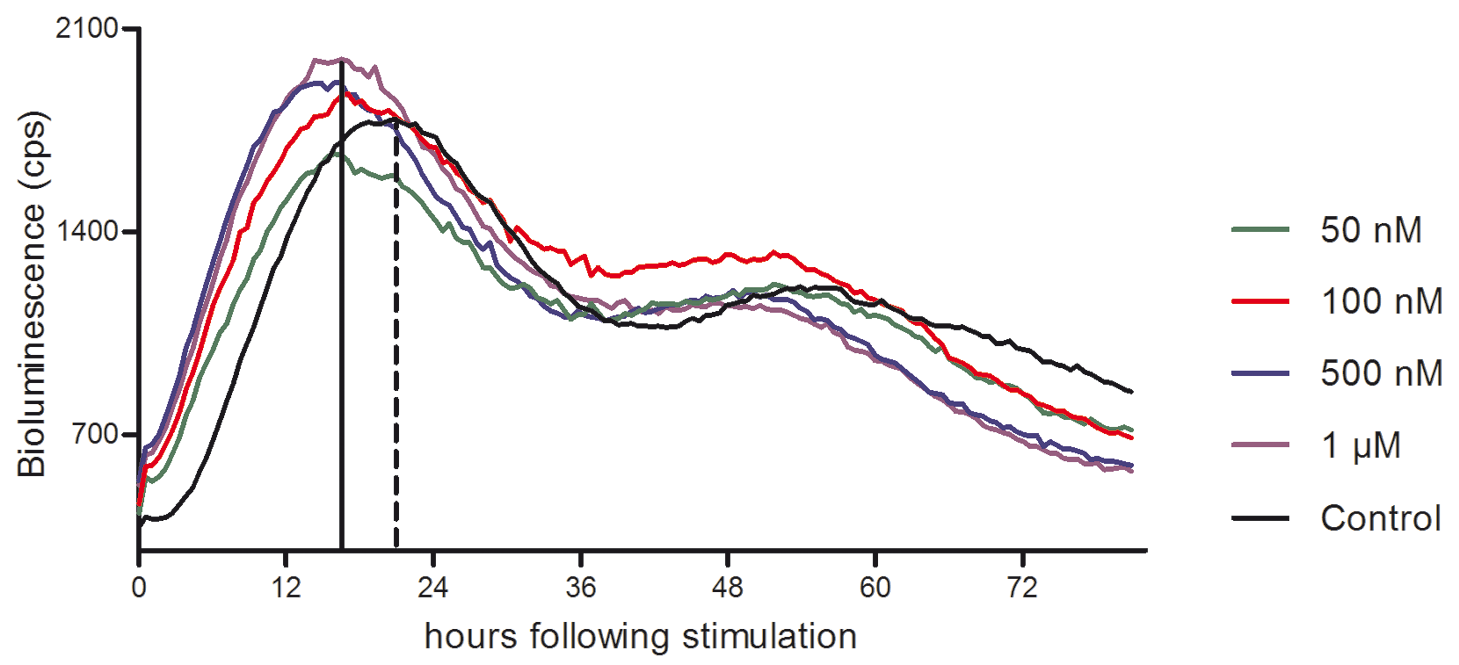

Supplement: Figure S5 — Effects of different dexamethasone doses to synchronize rhythmic clock gene expression. Bioluminescence (cps) of cavefish cells transfected with the zebrafish reporter construct, zfPer1b-Luc and transiently treated with different dexamethasone concentrations (50 nM, 100 nM, 500 nM, and 1 µM). A control lacking dexamethasone (“Control,” black trace) was also included. All 4 dexamethasone treatments were able to advance by 5±0.5 h the phase of a pre-existing oscillation evident in the control cells. Serum treatment during the seeding of the cells is responsible for the original establishment of this oscillation (unpublished data). The vertical dotted and solid lines indicate the peaks of the control and dexamethasone pulsed cells, respectively. (TIF) [file pbio.1001142.s005.tif]

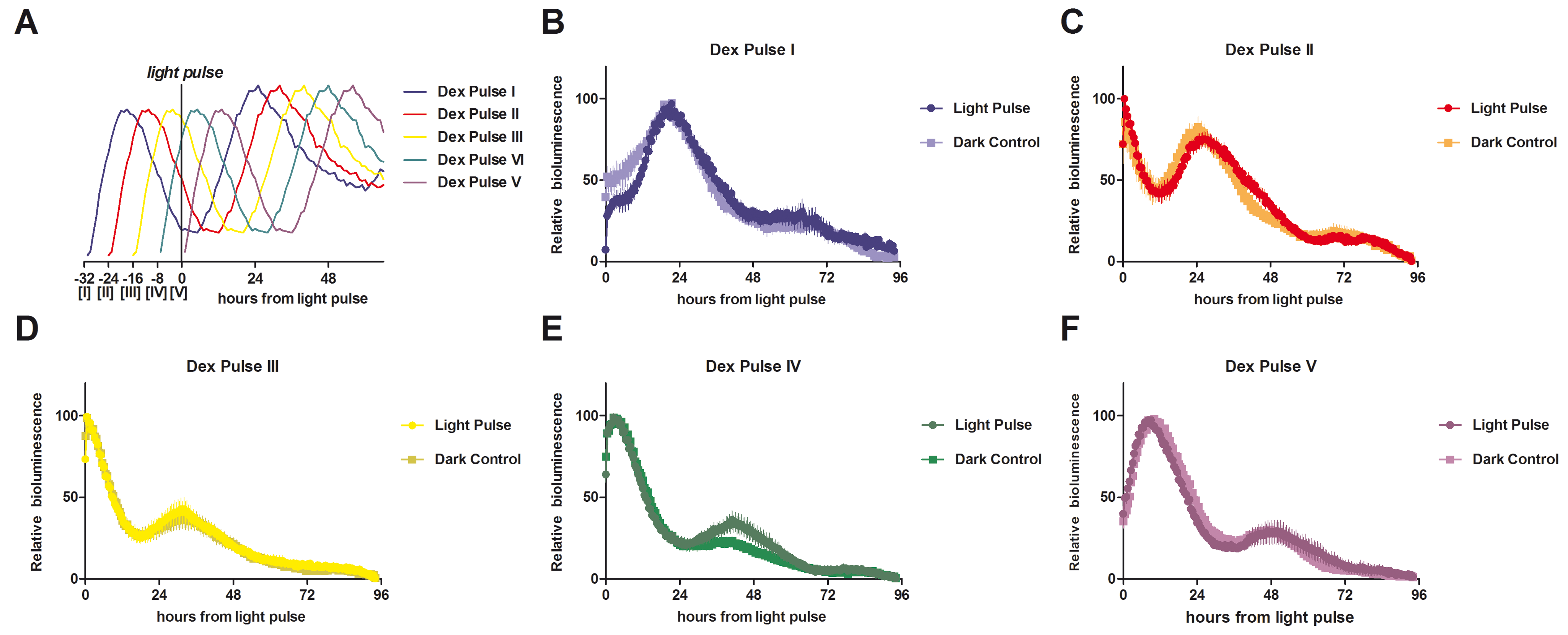

Supplement: Figure S6 — Light pulses fail to shift the phase of a dexamethasone-entrained cavefish clock. (A) Five sets of CF cells were transiently transfected with the zfPer1b-luc reporter and then at 8 h intervals (at time points I–V) were transiently treated with 100 nM dexamethasone before being simultaneously exposed to a 15 min light pulse. (B–F) Resulting bioluminescence profiles of the five sets of cells, compared with non-light-pulsed controls. At each time point, the mean ± SEM is plotted. During the assay, cells were maintained at a constant temperature and in constant darkness. In each panel, colours of the bioluminescence traces match those in the experimental design (A). Pale coloured traces represent constant dark controls, while dark coloured traces represent the light pulsed sets of cells (except D, where the light pulsed trace is shown as yellow and the dark control trace is shown as olive). None of the light pulsed sets of cells show a significant difference in phase relative to their constant dark controls (p>0.4). (TIF) [file pbio.1001142.s006.tif]

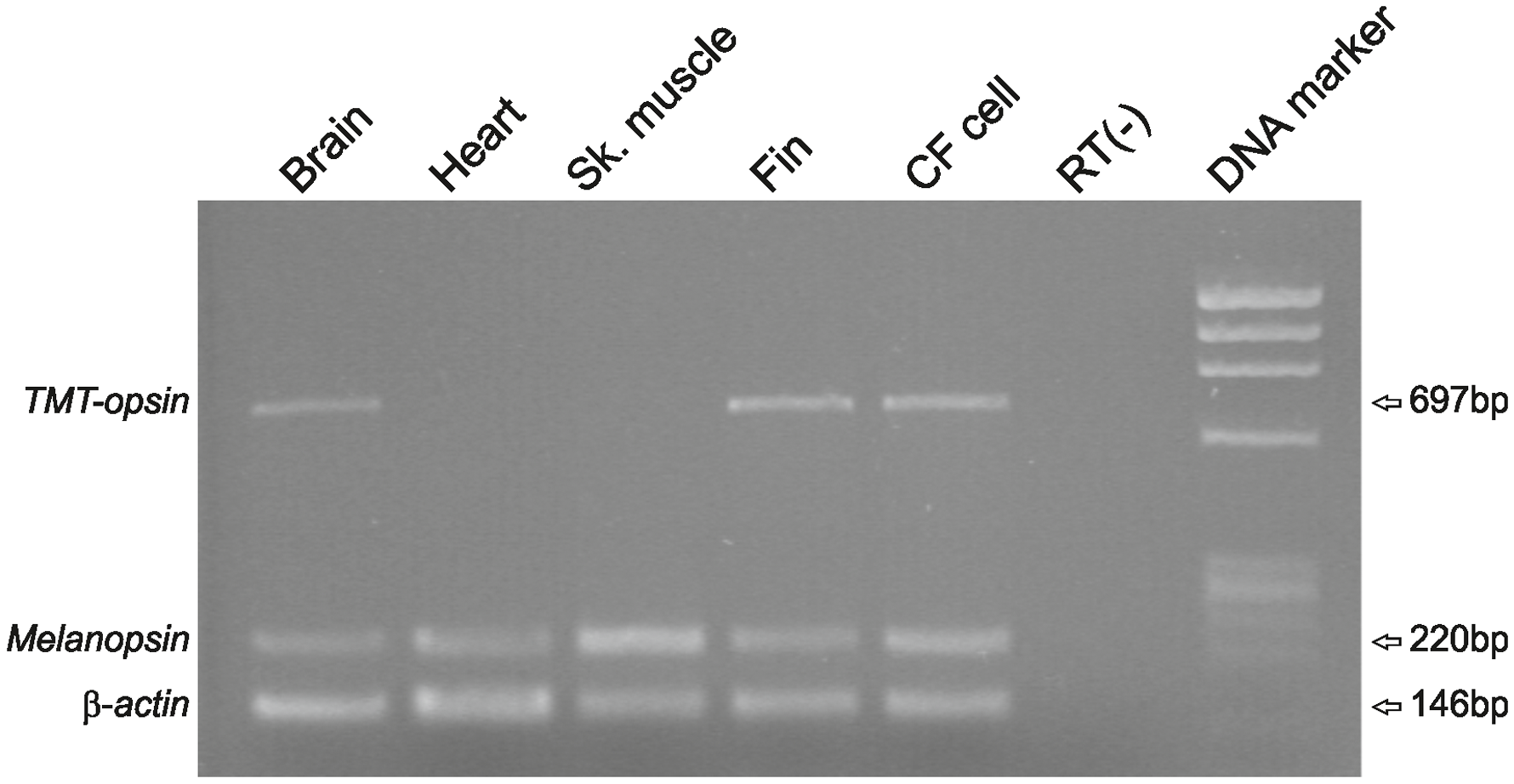

Supplement: Figure S7 — Tissue localization of Melanopsin (Opn4m2) and TMT-opsin in cavefish. RT-PCR results using brain, heart, muscle, fin, and CF cell RNA for the amplification of Melanopsin, TMT-opsin, and β-actin (as control). Equal volumes of the three PCR reactions performed for each tissue were subsequently mixed and then products were visualized by agarose gel electrophoresis. Melanopsin was expressed in all samples, whereas TMT-opsin was not found in the heart and the skeletal muscle (Sk. Muscle). A negative control lacking reverse transcriptase at the cDNA synthesis step was also included (RT (−)). (TIF) [file pbio.1001142.s007.tif]

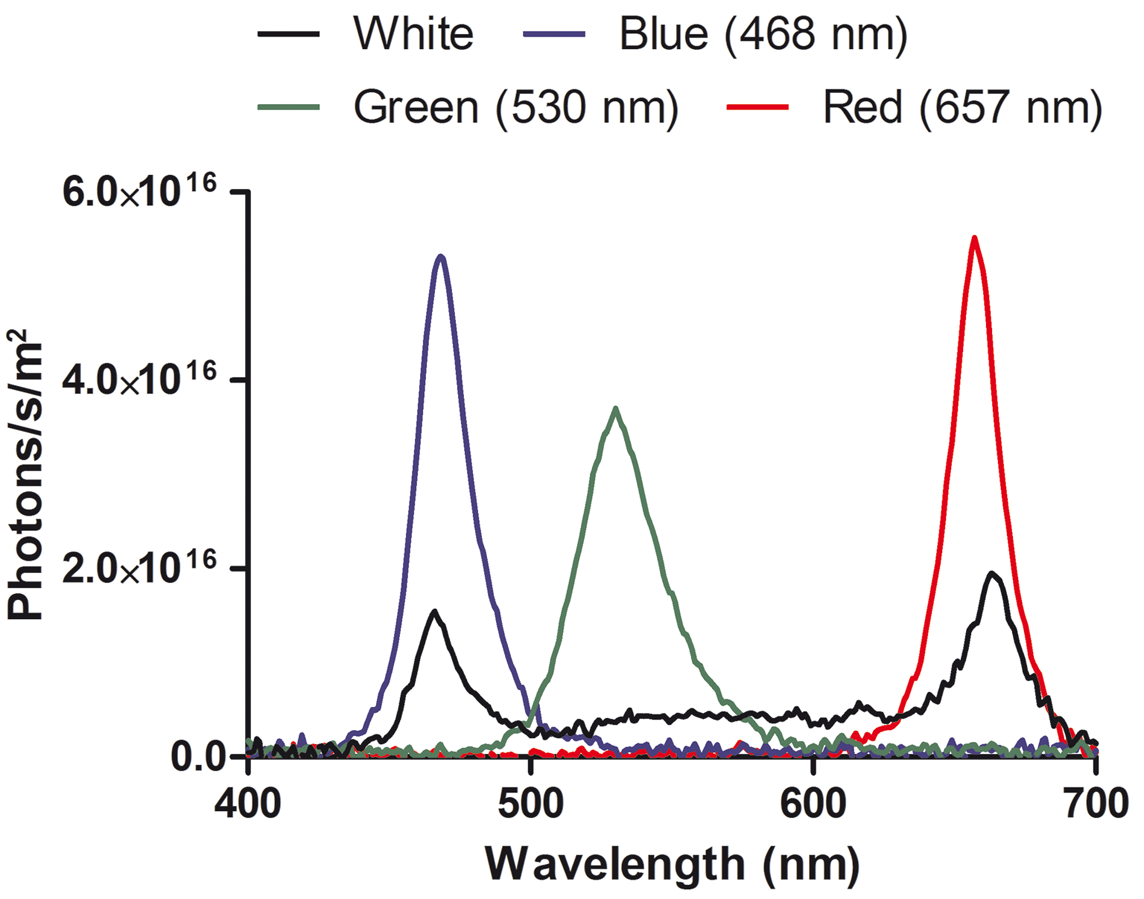

Supplement: Figure S8 — Irradiance curves for different monochromatic light sources. Light-emitting diode sources were used to produce white light (black trace) (450 nm<λ<700 nm) and monochromatic light in the blue (λpeak = 468 nm), green (λpeak = 530 nm), and red (λpeak = 657 nm) region of the spectrum. The light intensity of each source was adjusted to ensure that a constant number of photons was emitted by each source (1.42×10^18^±0.04×10^18^ photons/s/m2). (TIF) [file pbio.1001142.s008.tif]

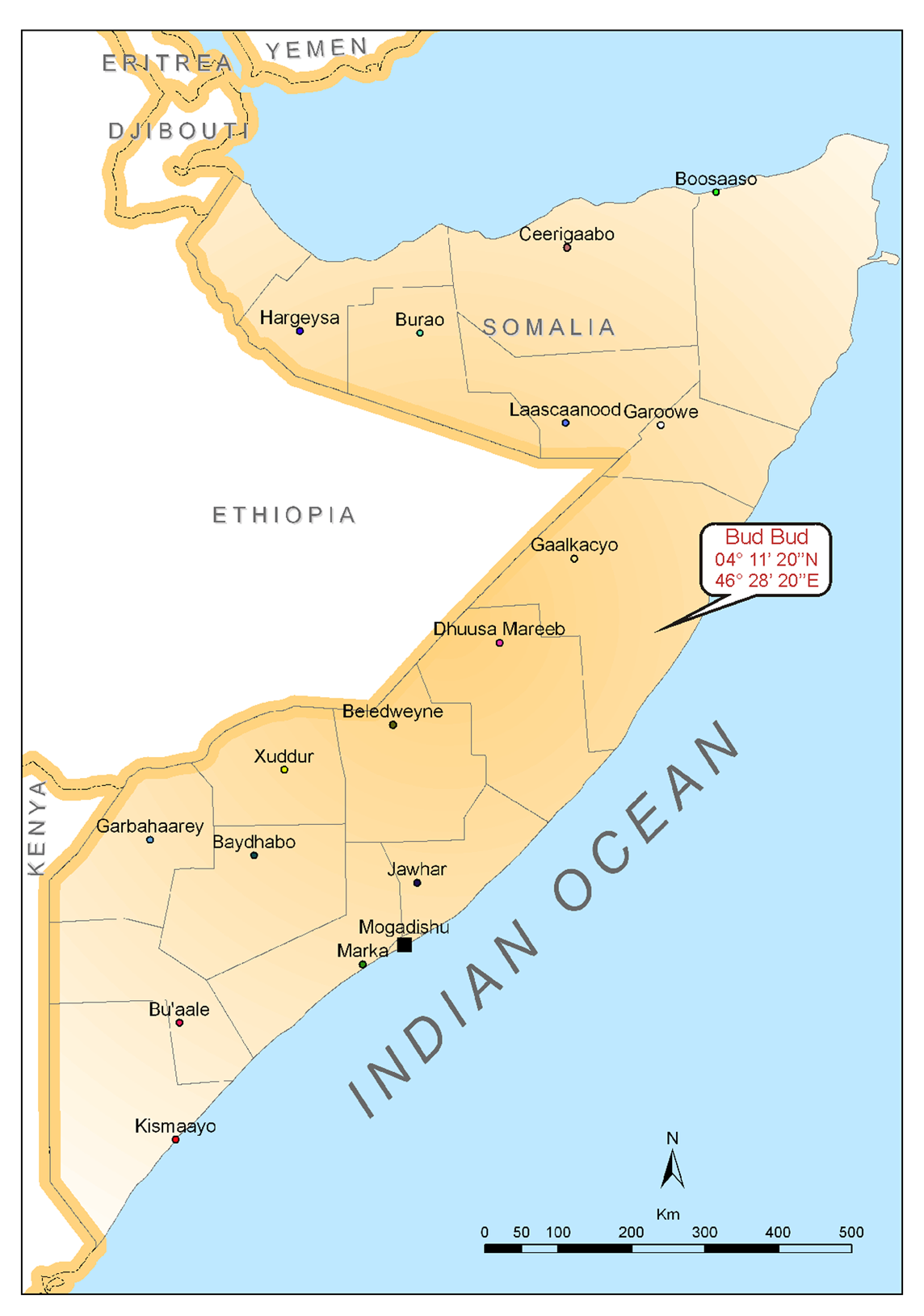

Supplement: Figure S9 — Origin of Phreatichthys andruzzii. Adult cavefish were originally collected in the wild in the oasis of Bud-Bud (04°11′19″N–46°28′27″E) in the centre of the Somalian desert during several expeditions to Africa (1968–1982). Ancestors of P. andruzzii entered the large phreatic layers of the Somalian desert that developed in Eocene horizontal limestone formations at the end of the Pliocene (1.4–2.6 million years ago) and became isolated with the extinction of epigean sister species as the result of extreme climatic changes. (TIF) [file pbio.1001142.s009.tif]

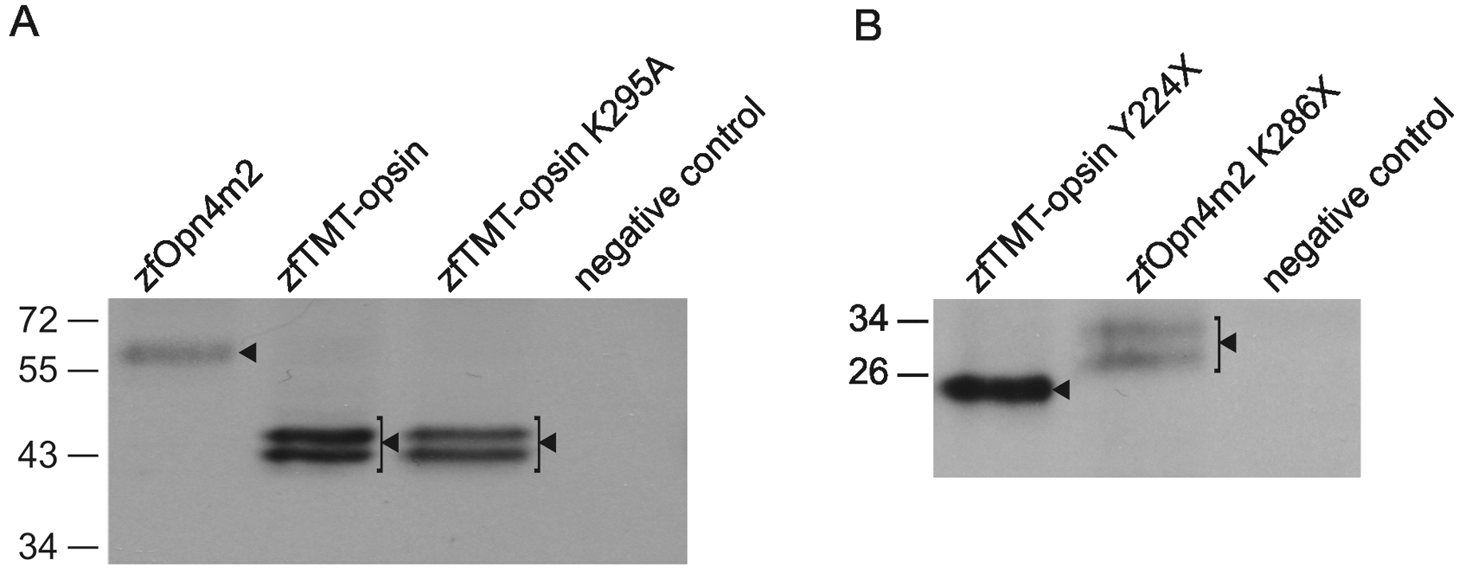

Supplement: Figure S10 — Expression of HA-tagged opsins in Hepa1-6 cells. Western blot analysis of cells transfected with HA-tagged expression vectors which encode (A) zfOpn4m2 (ca. 57 kDa), zfTMT-opsin (ca. 45 kDa), zfTMTK295A (ca. 45 kDa), and (B) zfTMT-opsinY224X (ca. 27 kDa) and zfOpn4m2K286X (ca. 34 kDa). The position of HA-immunoreactive opsin bands is indicated by arrowheads. The presence of doublet bands for some opsins may result from the denaturing conditions used to prepare the 7-transmembrane domain protein extracts for SDS PAGE analysis. Protein extracts prepared from cells transfected with the empty expression vector were loaded as negative controls. (TIF) [file pbio.1001142.s010.tif]
