# Supplementary material for: A Blind Circadian Clock in Cavefish Reveals that Opsins Mediate Peripheral Clock Photoreception
Source: PLoS Biol. 2011 Sep 6;9(9):e1001142. doi: 10.1371/journal.pbio.1001142 (PMC3167789; doi:10.1371/journal.pbio.1001142)
Supplement: Table S1 — PCR primers. Summary of the sequences of forward (F) and reverse (R) PCR primers used to amplify the initial P. andruzzii cDNA fragments (based on evolutionary conserved D. rerio nucleotide sequences) as well as the cavefish and zebrafish sequence primers used for quantitative PCR analysis. (DOC) [file pbio.1001142.s011.doc]

**PCR primers**

| **Gene** | **Primers for cloning cavefish genes** | **Quantitative PCR Primers (cavefish)** | **Quantitative PCR Primers (zebrafish)** |
| --- | --- | --- | --- |
| *Per1* | F: CTCCAGTGATCTGCTGGACCTGCT  R: TTGGCCATGAGGAGCCAGATG | F: GGCAACATCTCAACCAGTGG  R. GGGCTTCATAACCCGCGTGC | F: CCGTCAGTTTCGCTTTTCTC  R. ATGTGCAGGCTGTAGATCCC |
| *Per2* | F: ACCACAGGATTTAATCGGCA  R: GACGGTCTGGTCTTTGAAGG | F: TTCCAGCTGTGTGTTTCAGG  R: AGAAGCGGAAAGAGTGGTCA | F: ATGTCGATGGCTTTAGGCAG  R: CGAGACATCCAGAAGGTGCT |
| *Per3* | F: TGAACACACACTTAAAAACACGG  R: TAACATCTTCACACATGACTGGC |  |  |
| *Cry1a* | F: TGGTTCGCAGGATCCTCCAAC  R: TTCTCATCATGGTCATCAGA | F: TGATGAGAAGTTCGGGGTTC  R: GCGAATTGGCATTCATTCTT | F: TCCGCTGTGTGTACATCCTC  R: CAAACACTGCAGCAAAAACC |
| *Cry1b* | F: CAGATTCCATGGGACAGGAACCC  R: GCTGCACGAAAGCCACATCCAGCT |  |  |
| *Cry2a* | F: TACCCCAAACCCATGGTAAA  R: GCGATTTGCCGTTGTAACTT |  |  |
| *Cry2b* | F: ATGGAGGGAAACCCCATCTGCAT  R: CAGTAACAGTGGAAGAACTGCTGGAA |  |  |
| *Cry3* | F: TTGGATCGATGCCATTATGACCCAGCT  R: CCTCTGTAGTGTGAGAGCTGCTGGTA |  |  |
| *Cry4* | F: AAATGGAGAACCGCTCAGACGGG  R: GACAGCCACATCCAGTTCCC |  |  |
| *Cry5*  (*6-4 photolyase*) | F: CATGATAACCCTGCTCTG  R: TTGCACACCCAGTTCGTTCTCTCCAT | F: CTGCAGAGGTCCTTCCAAAG  R: GCTTTCCGTTGTTCTCTTCG | F: AATGGCAAGACTCCCATGAC  R: GTGGCCCTAAGGATGACGTA |
| *Clk1a* | F: ACCTCAGTTTATCAAGGAAATGTG  R: AACTCTGGCCTTGAGTTCCACTGGTGGTA | F: GTACTGTGGAGGAGCCCAAT  R: GGGTCTCCAGGTCATCCAC | F: CTGGAGGATCAGCTGGGTAG  R: CACACACAGGCACAGACACA |
| *Clk1b* | F: AAGAACTTGGGACCATGCTG  R: AGATGTGAATTCTTCATTGGGC |  |  |
| *Clk2* | F: GTTTGAGGTTTTGGGGACCT  R: GAGTCATGGCTGAATGCTGA | FOR: GGGACCTCGGGTTACGATTA  REV: TTCCTTTCCCACACTGCATC | FOR AGAACTGCTGAGGCTGCTGT  REV: TAACGTTGTGCTAGTCCCCT |
| *TMT-opsin* | F: TACAGCACCATGATGACCCCG  R: AGGTCTGGCGGACCGAAGGTG |  |  |
| *Opn4m2* | F: CCTGGAGACATCAACTGCACCGCA  R: CGTAGAGCTCACAAGGGCGCTC |  |  |
| *β-actin* | F: TATCCACGAGACCACCTTCAACTCCAT  R: AACGATGGATGGGCCAGACTCATCGTA | F: AGGACCTGTATGCCAACACA  R: AATCCACATCTGCTGGAAGG | F: GCCTGACGGACAGGTCAT  R: ACCGCAAGATTCCATACCC |
