# Supplementary material for: A Blind Circadian Clock in Cavefish Reveals that Opsins Mediate Peripheral Clock Photoreception
Source: PLoS Biol. 2011 Sep 6;9(9):e1001142. doi: 10.1371/journal.pbio.1001142 (PMC3167789; doi:10.1371/journal.pbio.1001142)
Supplement: Table S2 — P. andruzzii circadian clock cDNAs. Summary of the Genbank accession numbers for each of the P. andruzzii clock-related cDNAs cloned and sequenced. For each cDNA, the percentage of amino acid similarity with the zebrafish (D. rerio) homologs is also indicated. (DOC) [file pbio.1001142.s012.doc]

***P.andruzzii* circadian clock-related cDNAs**

| **Gene** | **Accession**  **no.** | **% of amino acid similarity with *D. rerio* homolog** |
| --- | --- | --- |
| *Cry1a* | GQ404476 | 96 |
| *Cry1b* | GQ404477 | 90 |
| *Cry2a* | GQ404478 | 95 |
| *Cry2b* | GQ404479 | 92 |
| *Cry3* | GQ404480 | 97 |
| *Cry4* | GQ404481 | 86 |
| *Cry5*  *(6-4 photolyase)* | GQ404482 | 89 |
| *Clk1a* | GQ404483 | 90 |
| *Clk1b* | GQ404484 | 91 |
| *Clk2* | GQ404485 | 92 |
| *Per1* | GQ404486 | 86 |
| *Per2* | GQ404487 | 82 |
| *Per3* | GQ404488 | 92 |
| *TMT-opsin* | GQ404490 | 88 |
| *Opn4m2* | GQ404489 | 86 |
| *β-actin* | GQ404475 | 100 |
